# Microbial exposure during early human development primes fetal immune cells

**DOI:** 10.1016/j.cell.2021.04.039

**Published:** 2021-06-24

**Authors:** Archita Mishra, Ghee Chuan Lai, Leong Jing Yao, Thet Tun Aung, Noam Shental, Aviva Rotter-Maskowitz, Edwin Shepherdson, Gurmit Singh Naranjan Singh, Rhea Pai, Adhika Shanti, Regina Men Men Wong, Andrea Lee, Costerwell Khyriem, Charles Antoine Dutertre, Svetoslav Chakarov, K.G. Srinivasan, Nurhidaya Binte Shadan, Xiao-Meng Zhang, Shabnam Khalilnezhad, Fabien Cottier, Alrina Shin Min Tan, Gillian Low, Phyllis Chen, Yiping Fan, Pei Xiang Hor, Avery Khoo May Lee, Mahesh Choolani, David Vermijlen, Ankur Sharma, Garold Fuks, Ravid Straussman, Norman Pavelka, Benoit Malleret, Naomi McGovern, Salvatore Albani, Jerry Kok Yen Chan, Florent Ginhoux

**Affiliations:** 1 Singapore Immunology Network (SIgN), A^∗^STAR, 8A Biomedical Grove, Immunos Building, Level 4, Singapore 138648, Singapore; 2 Translational Immunology Institute, Singhealth/Duke-NUS Academic Medical Centre, the Academia, 20 College Road, Discovery Tower Level 8, Singapore 169856, Singapore; 3 Department of Microbiology and Immunology, Immunology Translational Research Programme, Yong Loo Lin School of Medicine, Immunology Programme, Life Sciences Institute, National University of Singapore, Singapore 117597, Singapore; 4 Department of Mathematics and Computer Science, Open University of Israel, Ra’anana 4353701, Israel; 5 Department of Molecular Cell Biology, Weizmann Institute of Science, Rehovot 7610001, Israel; 6 Department of Reproductive Medicine, KK Women’s and Children’s Hospital, Singapore 229899, Singapore; 7 Genome Institute of Singapore (GIS), A^∗^STAR, 60 Biopolis Street, Singapore 138672, Singapore; 8 Program in Emerging Infectious Disease, Duke-NUS Medical School, 8 College Road, Singapore 169857, Singapore; 9 Experimental Fetal Medicine Group, Yong Loo Lin School of Medicine, National University of Singapore, Singapore 117597, Singapore; 10 Department of Obstetrics & Gynaecology, Yong Loo Lin School of Medicine, National University of Singapore, NUHS Tower Block, 1E Kent Ridge Road, Singapore 119228, Singpore; 11 Department of Pharmacotherapy and Pharmaceutics, Institute for Medical Immunology, ULB Center for Research in Immunology (U-CRI), Université Libre de Bruxelles (ULB), Brussels 1050, Belgium; 12 Harry Perkins Institute of Medical Research, QEII Medical Centre and Centre for Medical Research, the University of Western Australia, PO Box 7214, 6 Verdun Street, Nedlands, Perth, WA 6009, Australia; 13 Curtin Medical School, Curtin Health Innovation Research Institute, Curtin University, Perth, WA 6102, Australia; 14 Department of Physics of Complex Systems, Weizmann Institute of Science, Rehovot 7610001, Israel; 15 Department of Pathology and Centre for Trophoblast Research, Tennis Court Road, Cambridge CB2 1QP, UK; 16 OBGYN-Academic Clinical Program, Duke-NUS, Duke-NUS Medical School, 8 College Road, Singapore 169857, Singapore; 17 Cancer and Stem Cell Biology Program, Duke-NUS Graduate Medical School, Singapore 119077, Singapore; 18 Shanghai Institute of Immunology, Shanghai JiaoTong University School of Medicine, 280 South Chongqing Road, Shanghai 200025, China

**Keywords:** fetal Development, fetal immunity, microbes, bacteria, microbiome, immune priming, immune memory, Tem, Treg

## Abstract

The human fetal immune system begins to develop early during gestation; however, factors responsible for fetal immune-priming remain elusive. We explored potential exposure to microbial agents *in utero* and their contribution toward activation of memory T cells in fetal tissues. We profiled microbes across fetal organs using 16S rRNA gene sequencing and detected low but consistent microbial signal in fetal gut, skin, placenta, and lungs in the 2^nd^ trimester of gestation. We identified several live bacterial strains including *Staphylococcus* and *Lactobacillus* in fetal tissues, which induced *in vitro* activation of memory T cells in fetal mesenteric lymph node, supporting the role of microbial exposure in fetal immune-priming. Finally, using SEM and RNA-ISH, we visualized discrete localization of bacteria-like structures and eubacterial-RNA within 14^th^ weeks fetal gut lumen. These findings indicate selective presence of live microbes in fetal organs during the 2^nd^ trimester of gestation and have broader implications toward the establishment of immune competency and priming before birth.

## Highlights

• Human fetuses in 2^nd^ trimester show T cell diversity with effector-memory phenotype
• Fetal organs show diverse bacterial genera that can be cultured and propagated
• Bacterial structures with mucin-like threads are visualized in 14-weeks EGA fetal gut
• Fetal bacteria induce syngeneic memory T cell activation in fetal mLN T cells

## Graphical abstract

**Figure:**
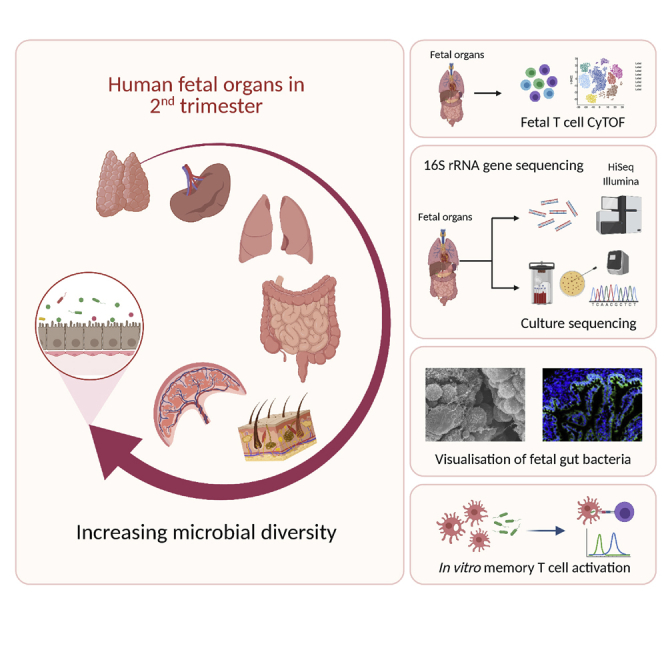

## Teaser

Analysis of human fetal tissues and the placenta in the 2^nd^ trimester of gestation identifies live bacterial strains that are able to induce the activation of memory T cells in the fetal mesenteric lymph node, thus providing insights into early life immunity.

## Introduction

The human fetal immune system is known to develop early during gestation with significant diversity in its immune repertoire and apparent sensitivity toward external antigens (Halkias et al., 2019; Li et al., 2019; McGovern et al., 2017; Papadopoulou et al., 2019; Park et al., 2020; Rackaityte and Halkias, 2020; Rechavi et al., 2015; Schreurs et al., 2019; Stras et al., 2019; Tieppo et al., 2020; Zhang et al., 2014), thus indicating a much more competent and diversified system than previously thought. We have shown previously that fetal dendritic cells (DCs) promote T cell-mediated immune suppression during early fetal development, indicating an active and responsive immune architecture during the 2^nd^ trimester of gestation (McGovern et al., 2017). However, the factors responsible for such *in utero* priming and their effect on the immune system of the developing fetus remain elusive.

Here, using a mass cytometry-based approach to study tissue-specific human fetal T cells across multiple organs, we identified cytotoxic T cells and most notably effector memory T cells (Tems) across the 2^nd^ trimester of gestation, in addition to regulatory T cells (Tregs). Tregs are known to be essential for the regulation of commensal microbe-specific effector and memory responses during homeostasis (Kuczma et al., 2020; Leech et al., 2019; Mold et al., 2010; Nutsch et al., 2016; Russler-Germain et al., 2017). Several studies have recently detected microbe traces in human placenta and fetal samples (Aagaard et al., 2014; Al Alam et al., 2020; Ardissone et al., 2014; Biasucci et al., 2008; Cao et al., 2014; Collado et al., 2016; Funkhouser and Bordenstein, 2013; de Goffau et al., 2019; Diaz Heijtz, 2016; Jiménez et al., 2005; Kundu et al., 2017; Parnell et al., 2017; Perez-Muñoz et al., 2017; Rackaityte et al., 2020; Romano-Keeler and Weitkamp, 2015; Seferovic et al., 2019; Stout et al., 2013; Willyard, 2018; Younge et al., 2019). However, active microbial presence *in utero* is still a topic of constant debate, and novel sensitive approaches are required to understand the complexities of human gestation (Collado et al., 2016; Fricke and Ravel, 2021; Perez-Muñoz et al., 2017; Robertson et al., 2019; Senn et al., 2020; Silverstein and Mysorekar, 2021; Stinson et al., 2019). Thus, we explored the presence of microbes in fetal tissues and their potential role in priming and activation of memory T cells during fetal development. Using 16S rRNA gene sequencing approach, we detected a low but consistent microbial signal across several fetal tissues in the 2^nd^ trimester of gestation with a significant increase in bacterial diversity compared to PBS controls. Sequencing results were validated by aerobic and anaerobic culturing of fetal tissues, followed by isolation and identification of several live bacterial strains. To visualize the bacteria in fetal tissues, we performed scanning electron microscopy (SEM) and RNA-*in situ* hybridization (RNA-ISH) and identified differentially located bacteria within the fetal intestinal lumen. Notably, fetal-isolated bacterial strains induced memory T cell activation and expansion of fetal lymph node T cells, thereby indicating a previous encounter and priming of fetal immune system with bacterial antigens. Altogether, our observations suggest a sparse microbial presence in fetal tissues and their potential priming of the fetal immune system, thereby leaving the footprints of microbial memory during early development. These findings have broad implications for our understanding of human development and the establishment of a competent and diverse immunity before birth.

## Results

### T cell pool during the 2^nd^ trimester of human fetal development is diverse and competent

**Figure S1:**
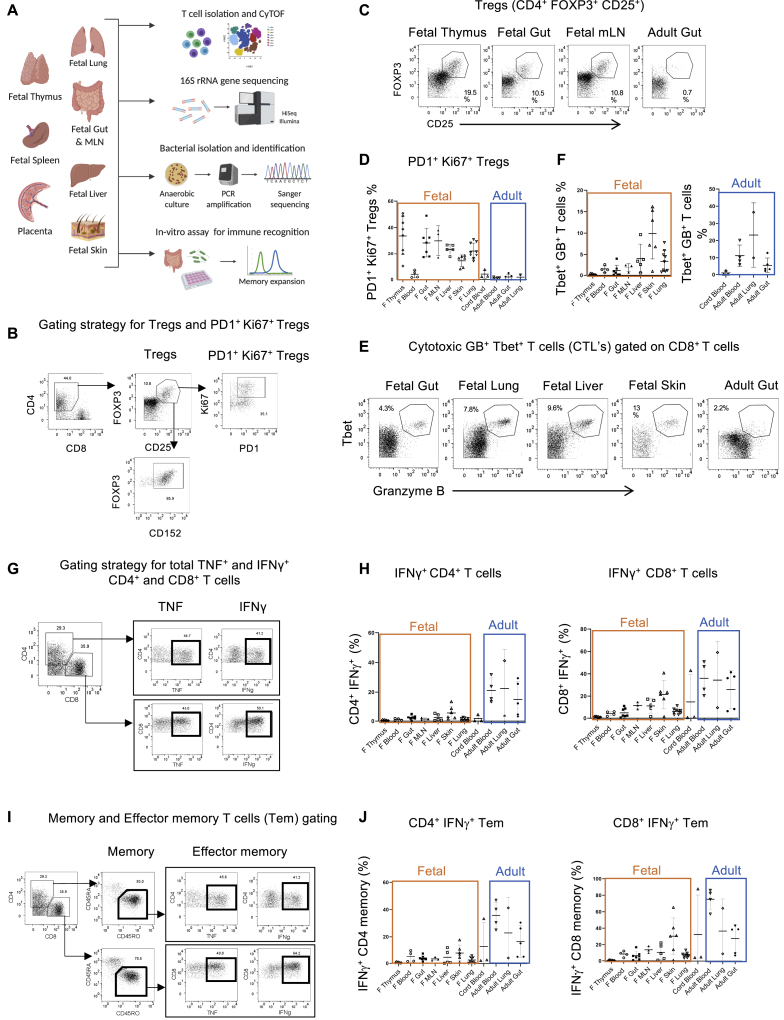
Study design and T cell diversity during the 2^nd^ trimester of human fetal development, related to Figure 1 (A) Schematic for introducing the study design and techniques used. Precisely, fetal tissues were harvested from the human fetuses under sterile conditions and were subjected to mass cytometry (CyTOF) for T cell identification, 16S rRNA gene sequencing for bacterial detection, anaerobic culture based bacterial propagation and in-vitro T cell expansion assay. (B) Flow cytometry plots and gating strategy for fetal and adult Tregs and PD1^+^ Ki67^+^ Tregs. CD25^+^ FOXP3^+^ Tregs were further checked for CD152 expression and more than 80% of Tregs were found to be CD152^+^ as well. (C) Representative flow cytometry plots for fetal and adult Tregs (D) Percentage of PD1^+^ Ki67^+^ Tregs in fetal versus adult tissues and in blood samples (fetal, adult and cord blood) as determined by mass cytometry analysis (input gating strategy and reference plots in Figure S1B). (E) Representative flow cytometry plots for cytotoxic T lymphocytes (CTL: CD8^+^ Tbet^+^ Granzyme B^+^ T cells) in fetal and adult samples. CTL’s are gated on total CD8^+^ T cells. (F) Percentage of cytotoxic T cells (CD8^+^ CTL) in fetal versus adult tissues and in blood samples (fetal, adult and cord blood) as determined by mass cytometry analysis. (G) Input gating strategy and representative flow-cytometry plots for identification of TNF^+^ and IFNγ^+^ cells in CD4^+^ and CD8^+^ T cells (H) Percentage of IFNγ^+^ cells in CD4^+^ (left) and CD8^+^ (right) T cells in fetal and adult tissues and in blood samples (fetal, adult and cord blood) as determined by mass cytometry analysis. (I) Input gating strategy and representative flow-cytometry plots for identification of CD4^+^/CD8^+^ CD45RO^+^ memory and effector memory T cells (Tem). For effector memory T cell phenotype identification, CD45RO^+^ memory T cells were gated and assessed analyzed for TNF and IFNγ expression as shown. (J) Percentage of IFNγ^+^ effector memory T cells (Tem) in CD4^+^ (left) and CD8^+^ (right) memory T cells in fetal and adult tissues and in blood samples (fetal, adult and cord blood) as determined by mass cytometry analysis.

To study the fetal immune system and its microbial components, we established a careful protocol for collection and processing of fetal tissues (see STAR Methods for detailed collection and processing protocol) (McGovern et al., 2017). The study is divided into four stages: T cell profiling by mass cytometry (CyTOF), 16S rRNA gene sequencing for bacterial identification, culture-based sequencing of live fetal bacteria, and *in vitro* T cell proliferation assay to demonstrate microbial immune recognition (Figure S1A). The samples collected were split between different arms of the study depending on the sample conditions and resource availability (Table S1).

**Figure 1:**
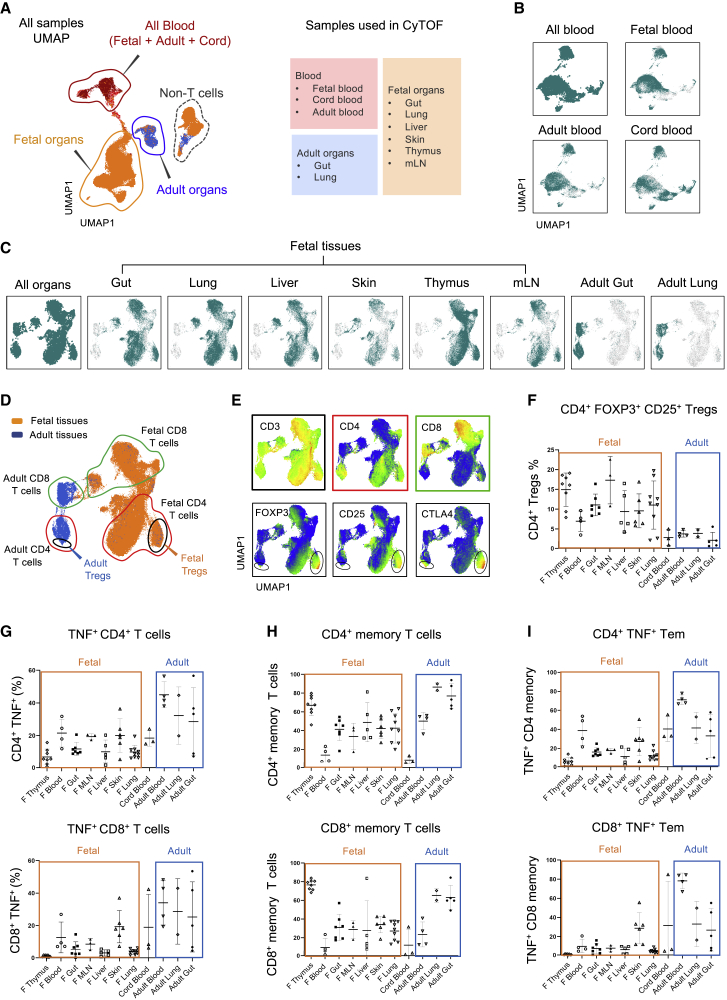
Identification of T cell diversity during the 2^nd^ trimester of human fetal development (A) Suspension mass cytometry (CyTOF) was performed on PMA/ionomycin-stimulated fetal T cells. The UMAP projection shows all the fetal and adult organs and blood samples used for T cell analysis: fetal gut (7), fetal lung (9), fetal liver (5), fetal skin (6), fetal thymus (8), fetal mLN (3), adult gut (5), adult lung (2), fetal (4), cord (3), and adult blood (4). Numbers in parenthesis represent number of samples being analyzed. (B) UMAP projection of all the blood samples, along with individual projections of each sample type (fetal, cord, and adult blood): fetal blood (4), cord blood (3), adult blood (4). (C) UMAP projection of the T cells from fetal and adult organs, along with individual projections of each organ over the UMAP: fetal gut (7), fetal lung (9), fetal liver (5), fetal skin (6), fetal thymus (8), fetal mLN (3), adult gut (5), and adult lung (2). (D) UMAP projection of fetal versus adult organs (in orange and blue, respectively). CD4^+^ and CD8^+^ T cell clustering is also shown on the same UMAP projection, in both fetal and adult tissues. Tregs in CD4 compartment is also shown based on CD4^+^CD25^+^FOXP3^+^CD152^+^ markers. (E) UMAP feature projection of T cell markers (CD3, CD4, and CD8) and Treg-associated markers (CD25, FOXP3, and CD152) are shown on top and bottom panels, respectively. (F) Percentage of Tregs in fetal versus adult tissues and in blood samples (fetal, adult, and cord blood) as determined by mass cytometry analysis (input gating strategy and reference plots in Figures S1B and S1C). As shown, Tregs are enriched in fetal organs as compared to adult organs and blood. (G) TNF cytokine expression in CD4^+^ (top) and CD8^+^ (bottom) T cells as determined by mass cytometry analysis of fetal and adult tissues (input gating strategy and reference plots in Figure S1G). (H) Percentage of CD4^+^ (top) and CD8^+^ (bottom) CD45RO^+^ memory T cells in fetal and adult tissues and blood samples (input gating strategy and reference plots in Figure S1I). (I) Percentage of TNF^+^ cells in CD4^+^ (top) and CD8^+^ (bottom) CD45RO^+^ effector memory T (Tem) cells in fetal and adult tissues and blood samples. See also Table S1.

We first profiled T cells from fetal lung, skin, gut, thymus, liver, mesenteric lymph nodes (mLN), and blood by using state-of-the-art CyTOF approach as recently described (Yeo et al., 2020) (antibody list in the Key resources table). Fetal spleens were not sufficiently large enough to use for both T cell analysis and bacterial DNA sequencing; hence, they were excluded from T cell profiling. Unsupervised uniform manifold approximation and projection (UMAP) analysis (Becht et al., 2018) of T cells from fetal (13–23 weeks estimated gestational age [EGA]) and adult samples revealed that fetal, adult, and cord blood clustered together at one end of the UMAP, whereas the fetal and adult organs formed two distinctly separate clusters (Figure 1A). This suggests organ-specific priming of T cells during the 2^nd^ trimester of gestation. A small fraction of non-T cells were also present, which were removed from further analysis. We next separated all blood samples from tissues followed by independent UMAP analysis for both datasets. The unsupervised clustering of all blood T cells further showed no distinct separation among fetal, adult, and cord blood samples based on our panel of markers (Figure 1B). However, the tissues consistently showed distinct separation between fetal and adult organs in UMAP analysis (Figure 1C).

We identified fetal- and adult-specific CD4^+^ and CD8^+^ T cells based on the marker projections as well as CD4^+^FOXP3^+^CD25^+^CTLA4^+^ Tregs, which formed distinct clusters for both fetal and adult organs (Figures 1D and 1E). The tight clustering of Treg populations from a range of fetal tissues indicates that Tregs have a conserved phenotype across organs. We observed a remarkable enrichment of Tregs in fetal tissues in comparison to adult organs (Figure 1F, Treg input gating strategy and reference plots in Figures S1B and S1C). Interestingly, fetal tissue Tregs were highly enriched for PD1^+^Ki67^+^ expression (Figure S1D), indicative of chronic TCR activation, active proliferation, and immune tolerance (Asano et al., 2017; Leung et al., 2018; Santegoets et al., 2014; Sharpe and Pauken, 2018). We also observed a consistent presence of CD8^+^Tbet^+^GB^+^ (granzyme B) cytotoxic T lymphocytes (CTL) in fetal tissues that were significantly enriched in fetal skin, lung, and liver (reference plots and quantification in Figures S1E and S1F).

Functional immune cells with potential antimicrobial activity and effector/memory phenotype have been demonstrated in fetal tissues (Haynes et al., 1988; Kumar et al., 2018; Li et al., 2018; Mackay and Gebhardt, 2013; McGovern et al., 2017; Schuster et al., 2009; Vermijlen et al., 2010). Thus, we probed our data for the expression of antimicrobial cytokines in CD4^+^ and CD8^+^ T cells (input gating strategy in Figure S1G). Notably, significant expression of tumor necrosis factor (TNF^+^) and interferon (IFN)γ^+^ T cells were observed across fetal tissues that were consistent among donors and tissue types (Figures 1G and S1H). We next explored the fetal T cell memory populations (CD4^+^/CD8^+^ and CD45RO^+^ T cells) across all 2^nd^ trimester fetal tissues. Interestingly, we observed a significant proportion of CD4^+^ and CD8^+^ memory T cells in fetal tissues, especially in thymus (Figures 1H, S1I, and S1J). Fetal organs like gut, mLN, lung, skin, and liver also had significantly high levels of memory T cells. We also observed the presence of TNF^+^ and IFNγ^+^ effector Tems in fetal tissues (Figures 1I and S1J). Importantly, the effector memory phenotype was observed in all fetal organs except thymus despite having significant memory T cells (Figures 1H and 1I). Together, these data indicate the presence of antigenic stimuli that drive the generation of activated T cells within the fetal organs.

### Identification of bacterial genera that colonize fetal tissues

**Figure S2:**
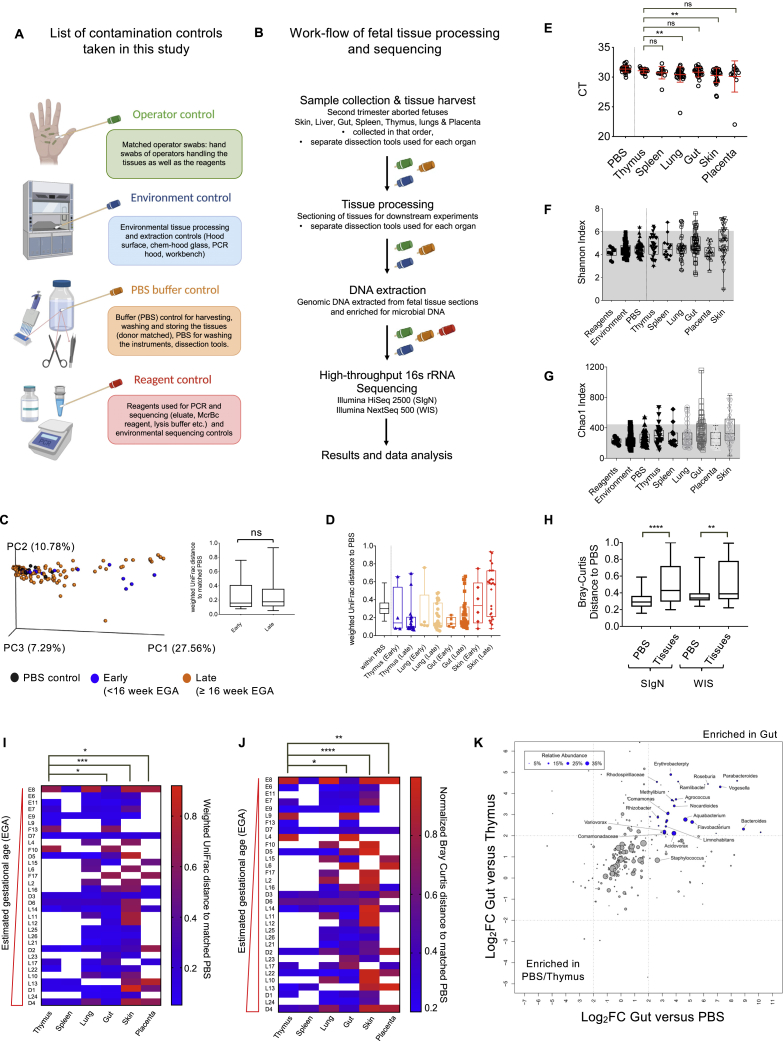
Contamination controls and high-throughput 16S rRNA gene sequencing of fetal tissues, related to Figure 2 (A) Schematic to show the contamination controls taken at each step ranging from fetal dissection to tissue processing, bacterial culturing, DNA extraction, and sequencing. The controls taken at each step can be divided into four major categories. Operator control includes the hand swabs of the operators handling the fetal tissues while dissection and processing and while DNA extraction. Environment control denotes control swabs taken from work surfaces like PCR hood, laminar flow, work bench etc. PBS buffer control denotes PBS used at each step of tissue processing and DNA extraction. Reagent control encompasses the molecular reagents used for DNA extraction thereby depicting the contaminations present in extraction kits and associated reagents. (B) Workflow employed for fetal tissue processing, dissection, DNA extraction and sequencing. Contamination controls taken at each step are denoted by swab-handles and color coded according to panel A (left). (C) Principal Coordinate (PC) analysis based on Weighted UniFrac distance to the PBS. PC analysis is this plot is shown in terms of early versus late estimated gestational age (ega). 2^nd^ trimester fetal samples were divided in two groups, early (< 16 weeks) and late (≥16 weeks) EGA, and color coded accordingly. The percentage of variance explained by the PC’s is indicated next to each axis. Weighted UniFrac distance of fetal tissues to their matched PBS controls, segregated by early (< 16 weeks) and late (≥16 weeks) EGA. Unpaired Mann-Whitney test: ^∗∗∗^p < 0.0005. (D) Similar analysis as in panel (B), further broken down into tissue types. Weighted UniFrac distances between all available PBS samples is also displayed as a reference (within PBS bar). Unpaired Mann-Whitney test: ^∗^p < 0.05; ^∗∗∗^p < 0.0005 (E) CT values for fetal organs plotted to determine the extent of bacterial DNA present in each sample. Lower CT value for a sample represents higher content of DNA being amplified. Here the CT values for each organ plotted and compared with those of those in fetal thymus. As shown, most of the fetal tissues had significantly higher microbial DNA content than fetal thymus. (F) Shannon index boxplot of samples, including reagent, environmental and PBS controls, and fetal tissues. The gray shade represents the cutoff value which is 95th percentile of all PBS samples (~6.324) and is used to perform the Fisher’s exact test in main Figure 2D. (G) Analysis similar to the panel E, except for Chao1 index used as the α-diversity metric. (H) Analysis similar to Figure 2E, except for Bray-Curtis distance used as the β-diversity metric (instead of Weighted UniFrac distance) (I) Unnormalized Weighted UniFrac distances of fetal tissues against their matched PBS controls. Analysis was restricted to fetuses with data available for ≥ 2 tissues. Paired Mann-Whitney test: ^∗^p < 0.05; ^∗∗^p < 0.005, ^∗∗∗^p < 0.0005. This analysis is similar to that in Figure 2F, except that ‘unnormalized’ Weighted UniFrac distances were used for β-diversity metric. (J) Bray Curtis distances of fetal tissues against their matched PBS controls. Analysis was restricted to fetuses with data available for ≥ 2 tissues. Distances were normalized within fetus, by dividing each value by the average Bray-Curtis distance within each fetus. Paired Mann-Whitney test: ^∗^p < 0.05; ^∗∗^p < 0.005, ^∗∗∗^p < 0.0005. (K) Relative abundance plot showing the significantly enriched taxa (log2FC > 2) in fetal Gut as compared to fetal thymus (internal control) and control PBS samples (external control). Each sphere represents differential taxa and the size of the sphere represents the percentage relative abundance of the given genera.

Recent studies have explored the presence of microbes or microbial DNA in the placenta, amniotic fluid, meconium, fetal lung, and fetal intestine (Aagaard et al., 2014; Al Alam et al., 2020; Ardissone et al., 2014; Biasucci et al., 2008; Cao et al., 2014; Chen et al., 2017; Collado et al., 2016; Funkhouser and Bordenstein, 2013; de Goffau et al., 2019; Diaz Heijtz, 2016; Hu et al., 2013; Jiménez et al., 2005; Kundu et al., 2017; Perez-Muñoz et al., 2017; Rackaityte et al., 2020; Romano-Keeler and Weitkamp, 2015; Willyard, 2018). However, the presence of live microbes in human fetus still remains debated. Our search for the potential stimuli at the source of the diverse immune response observed in fetal tissues led us to explore the presence of microbes in fetal tissues. Previously, we have shown that the human fetus has an active and responsive immune architecture during the 2^nd^ trimester of gestation (McGovern et al., 2017); hence this period was chosen for microbial investigation. We profiled multiple fetal organs, including gut, skin, lung, thymus, spleen, and placenta for the presence of bacterial signals using high-throughput 16S rRNA gene sequencing (sample information in Table S1 and primer sequences in Table S2). Given the high vulnerability for contaminations in such studies, and the new benchmarks introduced to account for reagent-associated contaminating microbes (de Goffau et al., 2018; Knight et al., 2018), we devised a stringent array of controls associated with each step in the workflow, ranging from tissue procurement to the sequencing run (Figures S2A and S2B). The controls taken in this study can be divided into four major categories: operator controls (hand-swabs taken from operators who have handled, dissected, and further processed the fetal samples); environment controls (swabs taken from all the surfaces that fetal tissues may have been exposed like work bench, PCR hood, etc.); PBS controls (fetus-matched buffers for each donor, and have undergone the same laboratory treatments as the fetal samples, from collection to sequencing); and reagent controls (including the molecular reagents used during DNA extraction and sequencing process such as elution buffer, McrBc reagent, etc.), which allowed us to monitor the contaminations present in extraction kits and accompanying reagents. All these controls allowed us to detect the contaminations introduced during organ collection and processing as well as by the reagents used in the process. We have also taken great care to reduce experimental bias and cross-contaminations by processing all samples in isolation, in a low-contaminant, controlled environment.

**Figure 2:**
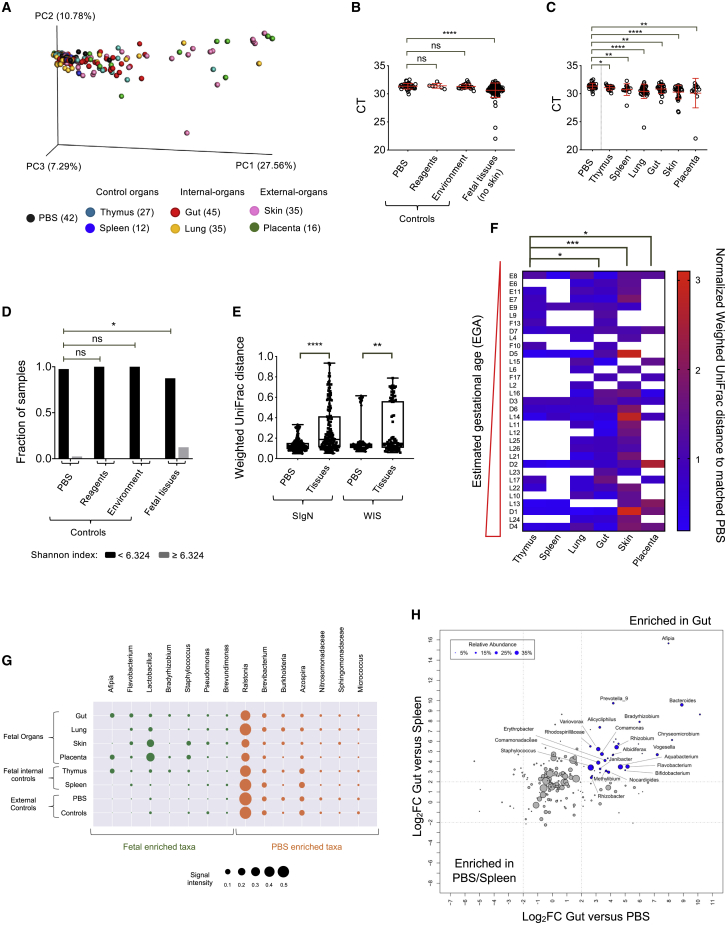
High-throughput 16S rRNA gene sequencing of fetal tissues and bacterial genera identification (A) Principal coordinate (PC) analysis based on weighted UniFrac distance to the PBS. The percentage of variance explained by the PC’s is indicated next to each axis. Each sample depicted by color and numbers in parenthesis indicate samples analyzed. (PERMANOVA R^2^ = 0.17741; p = 0.0009; analyzed using adonis function of vegan (v2.5.7) package in R). (B) Comparisons of bacterial 16S rRNA gene detection using qPCR. Comparison of CT (cycle threshold) values is shown along the y axis for each sample type plotted to determine the extent of bacterial DNA present in each sample. Unpaired Mann-Whitney test: ^∗∗∗∗^p < 0.0001. ns, not significant. (C) Comparison of CT values for each fetal organ against its PBS negative control. Unpaired Mann-Whitney test: ^∗^p < 0.05; ^∗∗^p < 0.005; ^∗∗∗∗^p < 0.0001. ns, not significant. (D) Fraction of samples above and below the 95^th^ percentile of Shannon indices in all PBS controls. A Fisher’s exact test was performed for each sample type against PBS controls (PBS, n = 42; reagents control, n = 10; environment, n = 55; fetal tissues, n = 169). ^∗^p < 0.05. ns, not significant. For reference to Shannon indices prediction see Figures S2F and S2G. (E) Weighted UniFrac distances between all PBS controls or between fetal tissues and their matched PBS controls, across two research facilities using two independent microbiome sequencing and analysis protocols: SIgN (Singapore Immunology Network) and WIS (Weizmann Institute of Science). Unpaired Mann-Whitney test: ^∗∗^p < 0.005; ^∗∗∗∗^p < 0.0001. ns, not significant. (F) Weighted UniFrac distances of fetal tissues against their matched PBS controls. Analysis was restricted to fetuses with data available for ≥2 tissues. Distances were normalized within fetus by dividing each value by the average weighted UniFrac distance within each fetus to determine β-diversity metric. Paired Mann-Whitney test: ^∗^p < 0.05; ^∗∗^p < 0.005, ^∗∗∗^p < 0.0005. (G) Dot-plot showing the distribution of bacterial genera as identified by OTUs across all sample types. Bacterial genera high in PBS and low or equal in samples are depicted as “PBS-enriched taxa” (orange, potential contaminants). Bacterial genera that were absent or very low in PBS and high in fetal tissues are denoted as “fetal-enriched taxa” (green, potential signals). Mean signal intensity of each genus was calculated and plotted using *matplotlib* (v.3.2.1) and *seaborn* (v.0.9.0) Python libraries. The samples are arranged in descending order of their signal strength in fetal gut. (H) Relative abundance plot showing the significantly enriched taxa (log2FC >2) in fetal gut as compared to fetal spleen (internal control) and control PBS samples (external control). Each sphere represents differential taxa and the size of the sphere represents the percentage relative abundance of the given genera. See also Tables S1, S2, S3, and S4.

Using the 16S rRNA gene sequencing data, we compared the overall microbial diversity between different fetal organs and their matched negative controls using weighted UniFrac distance matrices. The results of the β-diversity (diversity between fetal samples) were visualized by a principal coordinate analysis (PCoA) (Figure 2A). While negative control samples (PBS) mostly clustered on one end of the PCoA plot, fetal skin and placenta samples clustered on the opposite end, with few residing in-between. Fetal gut and lung samples were mostly dispersed between PBS and skin clusters, suggesting varying degrees of microbial diversity across tissues. PCoA plot annotated by early (<16 weeks) and late (≥16 weeks) fetal EGA across samples projected an unbiased generalized distribution across EGA, with no significant difference in early versus late gestational samples, when compared with matched PBS for bacterial diversity (Figures S2C and S2D). Most importantly, BactQuant (Liu et al., 2012) qPCR analysis across samples showed a significant increase in bacterial DNA in fetal tissues in comparison to the controls (PBS, reagents, and environmental controls) (Figure 2B). CT (cycle threshold) value represents the number of cycles required to amplify a threshold DNA signal, thereby lower CT value corresponds to higher content of starting DNA being amplified. Notably, this trend was consistent across tissues, with a significant increase in the microbial DNA signal in fetal organs when compared to PBS (Figure 2C) as an external control and fetal thymus as an internal control (Figure S2E). Consistent with this, a significant subset of fetal tissues displayed a within-sample microbial diversity (α-diversity) that was higher than the negative controls (Figures 2D, S2F, and S2G). Moreover, fetal tissue samples were significantly more dissimilar to their matched PBS controls (β-diversity) than the PBS samples were to each other (Figures 2E and S2H). The later result, in particular, was also reproduced in an independent set of fetal samples (Figures 2E and S2H), analyzed at a separate facility (Weizmann Institute of Science [WIS]) using dedicated experimental and analytical protocols tailored for low biomass microbiome studies (Geller et al., 2017; Nejman et al., 2020). Interestingly, we also noted a gradual increase in bacterial diversity in fetal tissues from external or surface-exposed organs in comparison to internal tissues (Figures 2F, S2I and S2J; 16S rRNA gene sequencing sample description and extended operational taxonomic units [OTU] information, in Table S3, sequencing reads for samples at separate facility [WIS] are in Table S4). These observations also corroborate with our findings of TNF^+^ T cells (Figures 1G and 1I) and cytotoxic CD8^+^ T cells (Figures S1F, S1H, and S1J) being more abundant within fetal skin than other organs. Together, these findings indicate the presence of an above-background microbial signal in at least some of the analyzed fetal tissues, albeit in very low abundance.

Despite having an extensive array of controls, we are aware that contaminations may still occur; hence we attempted to compare the bacterial taxa present in control samples to those in fetal tissues. We analyzed our 16S rRNA gene sequencing data based on its operational taxonomic classification using the SILVA (v.123) database (Quast et al., 2013). We identified the specific OTU associated with bacterial genera present in each sample (Figure 2G). Showing the average abundance of dominant bacterial taxa identified in PBS versus those in fetal tissues, our analysis highlighted the enrichment of 7 bacterial genera specifically enriched in fetal samples (denoted as “fetal-enriched taxa” in green), including *Flavobacterium*, *Lactobacillus*, *Staphylococcus*, *Afipia*, *Pseudomonas*, *Bradyrhizobium*, and *Brevundimonas.* We also identified 7 bacterial genera enriched in PBS and other controls (operator, reagent, and environmental controls; denoted as “PBS-enriched taxa” in orange) including *Ralstonia*, *Brevibacterium*, *Burkholderia*, *Azospira*, *Nitrosomonadaceae*, *Sphingomonadaceae*, and *Micrococcus* (Figure 2G; Table S3 showing the OTU information for each sample). Interestingly, each one of the fetal-enriched genera showed a heterogeneous distribution among tissues, suggesting a true signal coming from specific organs. On the contrary, the PBS-enriched genera showed a more homogeneous distribution across samples in both controls and tissues indicative of a systemic contamination. In addition, our comparative analysis of bacterial enrichment in fetal gut samples in comparison to fetal spleen (or thymus) versus PBS control showed similar pattern with ∼22 significantly enriched taxa in fetal gut (Figures 2H and S2K). These observations suggest significant enrichment of bacterial signals in fetal organs, more notably in fetal gut.

### Bacteria from freshly harvested fetal samples are viable and can be cultured

**Figure 3:**
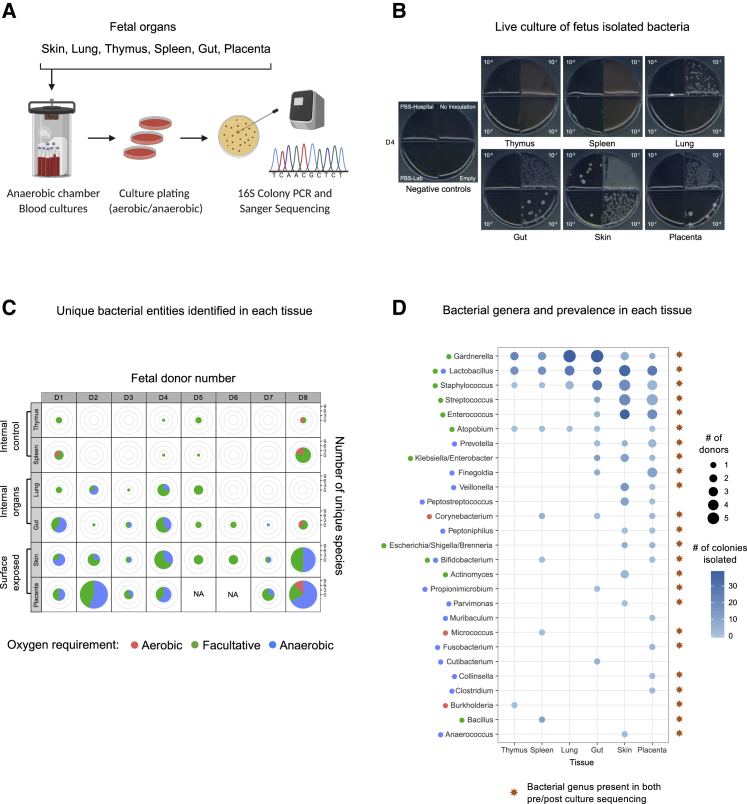
Culture and isolation of live bacteria from freshly harvested fetal samples (A) Schematic representation for anaerobic culturing of fetal bacteria, followed by colony selection, PCR, and Sanger sequencing of the isolates. (B) Plate images of serially diluted cultures of fetus-matched tissue inoculates (D4 donor). Negative controls include PBS samples used at hospitals to clean surgical equipment during harvesting and tissue dissection (PBS-Hospital) and PBS samples used in the laboratory to clean tools used during tissue biopsy (PBS-Lab). (C) Number of unique bacteria species identified from each fetal tissue inoculate. Data is combined from plates incubated under aerobic and anaerobic conditions. The proportion of aerobes, facultative anaerobes, and obligate anaerobes was determined from available literature. (D) Dot-plots representing the bacterial genera isolated and identified from fetal samples and their prevalence across fetuses and tissues. Colored dots next to the names of each genus indicate the oxygen requirement of known species with that genus (color coding same as in C). Multiple dots reflect variability within the genus with respect to their oxygen requirements. The orange star at the right side of the plot represents the bacterial genera that were identified by both pre-culture and post-culture sequencing for the same tissues (for reference see Figure S3D). See also Tables S1, S2, S3, S5, and S6.

**Figure S3:**
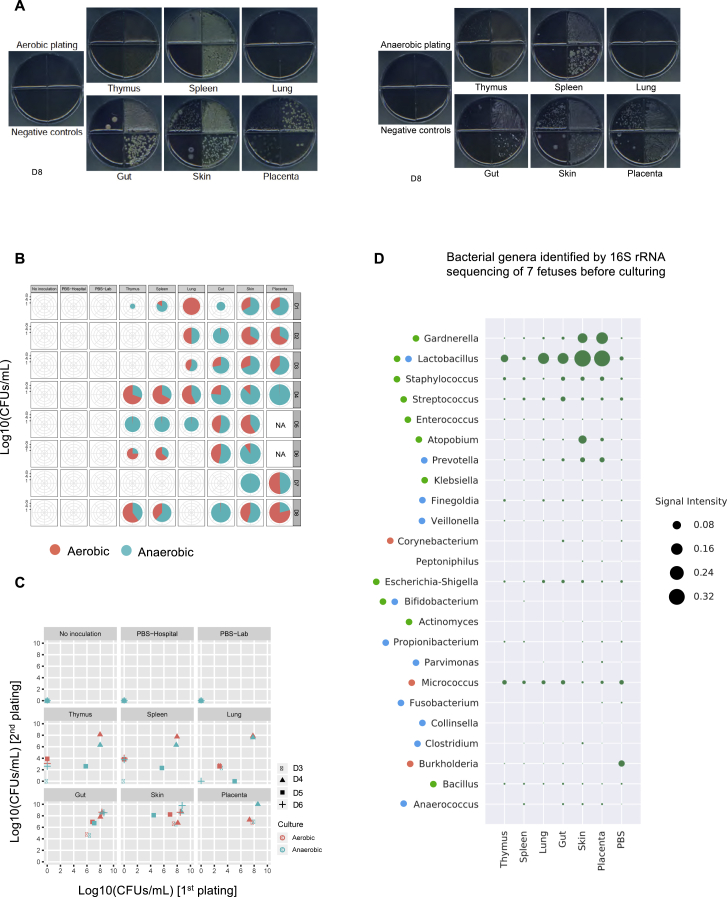
Plating specificity, efficiency, and reproducibility of fetus-associated bacteria, related to Figure 3 (A) Plate images of serially diluted cultures of fetus-matched tissue inoculates from a separate donor compared to main Figure 3B (D8), plated under either aerobic (left) or anaerobic conditions (right). All negative controls (plated as in Figure 3) were clean under both plating conditions, while the colony morphology, color and size across different plating conditions had notable differences even within the same fetal tissue inoculate. (B) Plating efficiency (expressed as CFUs/mL) of fetal tissue cultures under aerobic and anaerobic plating conditions across donors and tissues. The area of each pie chart is proportional to the sum of the CFUs/mL values obtained in the two growth conditions. (C) Reproducibility of CFU/mL measurements of selected fetus-matched tissue cultures under aerobic and anaerobic plating conditions, between two independent culture experiments. (D) Dot-plot showing the distribution of fetal genera present across 7 out of 8 donors which were simultaneously sequenced before (and after) culturing by high-throughput 16S rRNA sequencing. This analysis is complementary to that in main Figure 3D where the same fetal samples were sequenced after culturing for 48 hours. Mean signal intensity of each genus was calculated and plotted using *matplotlib* (v.3.2.1) and *seaborn* (v.0.9.0) Python libraries. For main Figure 3D, matched PBS were not sequenced post-culturing since there were no detectable colonies in PBS samples. While for pre-culturing, as shown we also sequenced PBS samples, for comparisons.

To determine if these DNA signatures were predictive of the presence of live bacteria, we inoculated tissue biopsies of freshly harvested fetal organs (from a separate cohort of 8 fetuses D1–D8 shown in Tables S1 and S3) in pre-reduced anaerobic blood-supplemented basal media under anaerobic conditions and plated the cultures under either aerobic or anaerobic conditions (Figure 3A; see STAR Methods for details). Several negative controls were included in each of these experiments, including PBS samples used at the hospital to clean surgical equipment during fetal dissection and PBS samples used in the laboratory to clean tools used during biopsy. None of these associated controls produced detectable colonies for any of the 8 distinct fetuses (Figures 3B, S3A, and S3B). In contrast, most of the cultured fetal tissues (6 out of 8 fetal donors) produced microbial colonies under both aerobic and anaerobic conditions (Figure S3B). Independent cultures and plating experiments produced reproducible numbers of colonies (Figure S3C). Moreover, we observed that plates carrying lung isolates appeared more oxidized, potentially reflecting a higher hemolytic ability of lung-associated bacteria. We also observed that the bacterial colony morphologies and sizes varied among different tissues suggesting a bacterial heterogeneity across organs. The absence of microbial growth from the negative controls and the apparent phenotypic differences in bacterial colonies across tissues are against the possibility of a common contamination by a single or few exogenous bacterial species. We next isolated and purified several colonies per inoculate and identified the bacterial genus via colony PCR and standard 16S sequencing (Figure 3C). Consistent with our high-throughput 16S sequencing results, a higher number of unique bacterial genera were identified in placenta or skin than in lung or gut samples and were further reduced in the spleen and thymus, consistent with a decreasing gradient of microbial diversity from external or surface-exposed fetal organs to internal organs (Figures 3C, S3B, and S3C). A large fraction of the isolated species is reported as facultative anaerobes or obligate anaerobes (Figures 3C, S3B, and S3C**;** Table S5). This is an interesting observation, because the oxygen supply to the fetus is reported to be dependent on the partial oxygen pressure gradient between maternal blood and fetal organs, which is low in early gestation and increases gradually along the gestational timeline (Gitto et al., 2002; Torres-Cuevas et al., 2017). We next surveyed the prevalence of bacterial genera across donors and fetal organs (Figure 3D). Although *Gardnerella*, *Lactobacillus*, and *Staphylococcus* were isolated from most of the fetal organs, other commonly isolated genera included *Streptococcus*, *Enterococcus*, and *Prevotella*. Importantly, we observed several bacterial genera, which were both strict anaerobes and are not previously reported as vaginal or skin commensals, such as *Bifidobacterium spp*., *Collinsella aerofaciens*, or *Clostridium hathewayi* (re-classified as *Hungatella Hathewayi*) (Kaur et al., 2014; Table S6 shows number of tissues positive for the given bacteria and number of colonies isolated for the given bacteria across 8 fetal donors). Interestingly, many of these cultivatable bacteria were also found in our 16S rRNA gene sequencing data (Figure 2G), where *Lactobacillus* and *Staphylococcus* were among the top fetal-enriched taxa whereas *Micrococcus* and *Burkholderia* were among the top PBS-enriched taxa.

To address the possibility of contamination during culturing, we also sequenced a section of the tissue biopsies before culturing from 7 of the 8 donors, using high-throughput 16S rRNA gene sequencing. While matching this pre-culture sequencing data with the culture-based sequencing results from the same biopsies, we successfully identified 24 out of 27 distinct bacterial genera (by their respective OTU) (Figure S3D) that were enriched in both datasets (Figure 3D, orange stars represent matching 24 genera present in both pre- and post-culture sequencing data). Notably, *Lactobacillus*, *Staphylococcus*, *Streptococcus*, *Enterococcus*, *Bifidobacterium*, *Prevotella*, and *Finegoldia* were among the ones identified by both pre- and post-culture sequencing, hence denoting a true signal from fetal tissues, and are not introduced due to culturing (Table S3 contains the extended sample and OTU information for the given 7 fetal donors used in Figure S3D). We also observed that a majority of these bacteria were either absent or very low in PBS samples when compared to the fetal organs. In contrast, *Micrococcus*, a potential contaminant that was consistently present in all samples including control PBS in our data, was recently reported to be present in fetal intestine *in utero* (Rackaityte et al., 2020). Similarly, *Streptococcus* that we isolated in our culture-based method has also been previously associated with human placental tissues, although it is speculated that it might be as a result of a potential infection (de Goffau et al., 2019). Therefore, the notion of true fetal-specific bacteria still remains open and demands further investigation. Taken together, our results reveal a sparse microbial presence in certain fetal tissues, some of which can be propagated by anaerobic culturing. However, their abundance in specific fetal tissues and their prevalence across gestational timeline still remains debatable and an area of active exploration. Neverthelss, these results indicate a transient presence of microbial entities within fetal tissues, which may remain in a constant flux, hence reflective of a snapshot in time.

### Bacteria-like morphologies and eubacterial RNA can be visualized in fetal gut lumen

**Figure 4:**
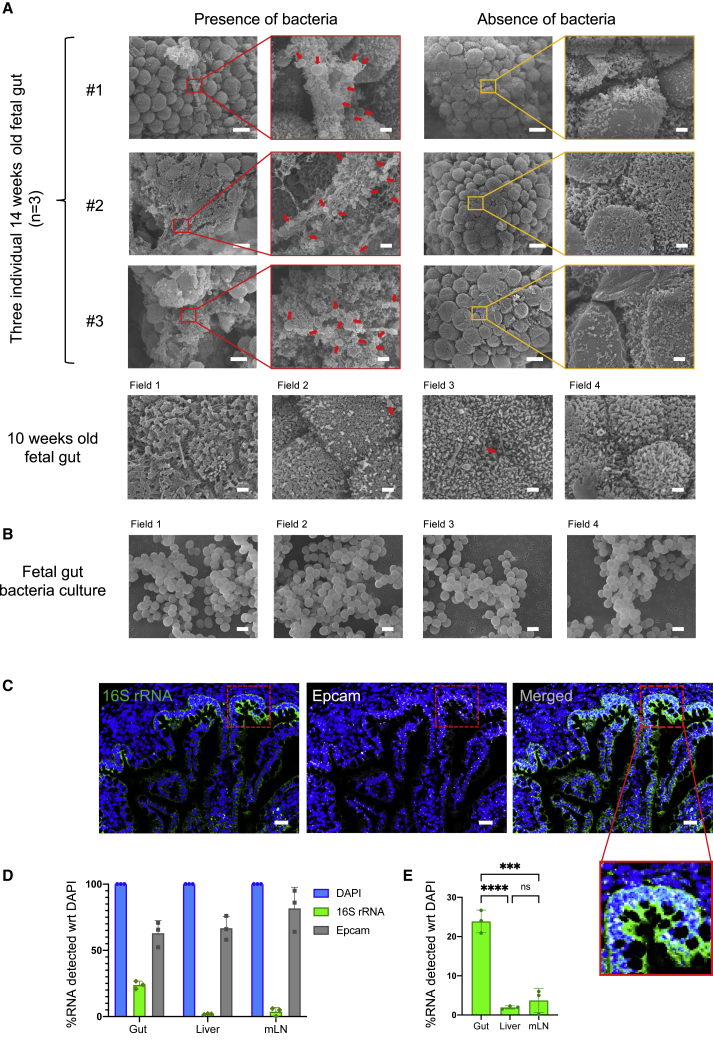
Visualization of bacterial structures in fetal gut lumen using SEM and RNA-ISH (A) Representative SEM images of 14-weeks EGA fetal mid-gut from three individual fetal samples (n = 3) showing the mucosal area with bacteria (left) and area devoid of bacteria (right) in low and high magnification images. One 10-weeks EGA fetal mid-gut lumen is also shown below with no detectable bacterial structures. Red arrowheads indicate bacteria-like structures within the intestinal lumen. Cocci-like bacteria were seen with an average size of 1 μm in all the three 14-weeks EGA fetal mid-gut lumens. Scale bars, 10 μm for low magnified views and 1 μm for high magnified views. (B) SEM images of cultured bacteria (isolated from 18-weeks EGA fetal gut) attached on the nitrocellulose membrane. Cocci-like bacterial structures were prominently seen with an average size of 1 μm. Scale bar, 1 μm. (C) Representative RNA-*in situ* hybridization (RNA-ISH) images of fetal gut lumen demonstrating the pan-bacterial probe against bacterial 16S rRNA in green and eukaryotic epithelial cell marker Epcam-specific RNA probe in white. Image on the right shows merged file and the image in inset represents zoomed-in view to visualize the bacterial RNA along-side epithelial boundaries within the lumen of fetal intestine (mid-gut). Scale bar, 40 μm. (D) Bar plot representation of EuBac 16S rRNA and Epcam signal quantification with respect to DAPI signal at 100% saturation (n = 3). The fetal liver and mLN samples were taken as controls. (E) Bar plot representation of EuBac 16S rRNA signal quantification in fetal mid-gut lumen beside fetal liver and mLN samples. Significant detection of bacterial 16S rRNA found in fetal gut lumen (n = 3). Statistical significance between experimental groups was determined by two-tailed, unpaired Student’s t test (^∗∗∗∗^p < 0.0001, ^∗∗∗^p < 0.001, ^∗∗^p < 0.01). See also Figure S4.

**Figure S4:**
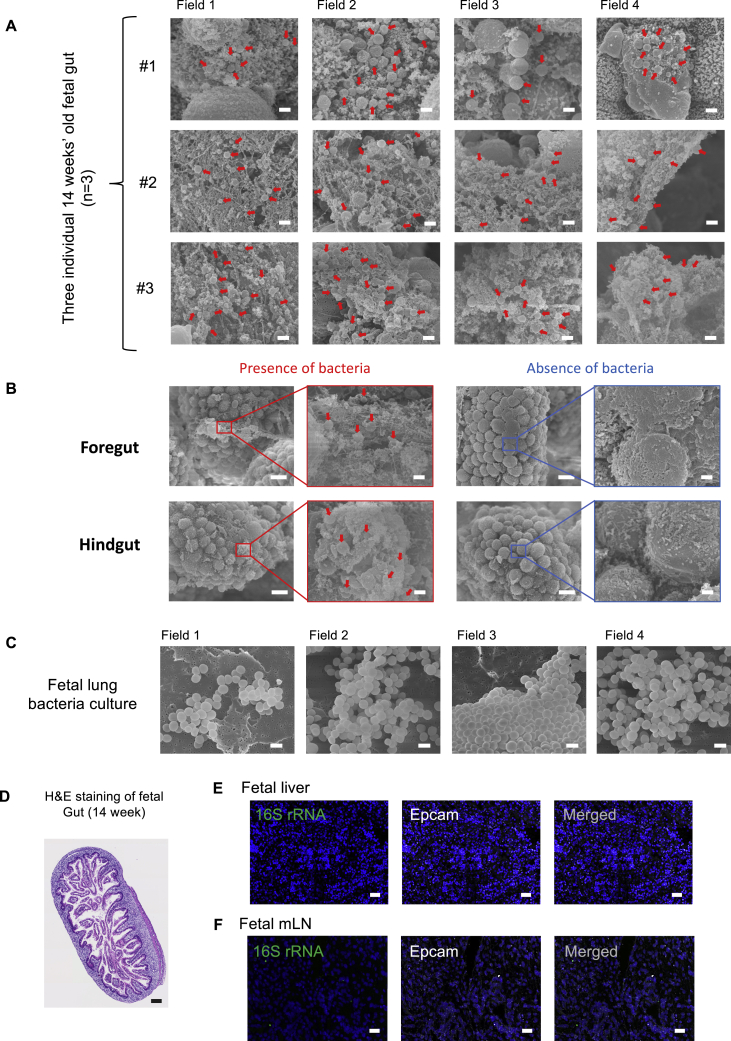
Bacterial structure visualization in fetal gut lumen using scanning electron microscopy, related to Figure 4 (A) Representative SEM images of 14 weeks old EGA fetal mid-gut from three individual fetal samples (n = 3) from multiple fields. Red arrowheads indicate bacteria-like morphologies within the intestinal lumen. Cocci like bacteria were embedded in the mesh-like mucus covering the intestinal cells. Red arrows indicate bacteria-like morphologies. (Scale bars = 1 μm) (B) EM images of 14 weeks old fetal foregut (top) and hindgut (bottom) showing the mucosal area with bacteria and mucosal area devoid of bacteria from one fetus (14 weeks EGA). Cocci bacteria (average size of 1 μm) were observed and there were also presence of cocci like bacterial structures found in 14 weeks’ old fetal foregut and hindgut. Red arrows indicate bacteria-like morphologies. (Scale bars; 10 μm for low magnified views and 1 μm for high magnified views) (C) SEM images of cultured bacteria (isolated from 18 weeks old fetal Lung) attached on the nitrocellulose membrane. Cocci-like bacterial structures were prominently seen with an average size of 1 μm. (Scale bar; 1 μm). (D) Representative image of Hematoxylin & eosin stain (H&E staining) of fetal mid-gut cross-section (isolated from 18 weeks old fetal gut), from serial sections used for RNA-ISH imaging. The tissue were embedded in FFPE blocks prior to slide preparation. 200 μm magnification. (E) Representative RNA-ISH imaging (RNAscope) images of fetal liver demonstrating the pan-bacterial probe against bacterial 16S rRNA in green and eukaryotic epithelial cell marker Epcam specific RNA probe in white. Image on the right shows the merged file. There were no significant spots detected for bacterial 16S rRNA in fetal liver. Scale bar, 40 μm. (F) Representative RNA-ISH imaging (RNAscope) images of fetal mesenteric Lymph Nodes (mLN) demonstrating the pan-bacterial probe against bacterial 16S rRNA in green and eukaryotic epithelial cell marker Epcam specific RNA probe in white. Image on the right shows the merged file. There were no significant spots detected for bacterial 16S rRNA in fetal mesenteric lymph nodes. Scale bar, 40 μm.

To investigate the direct presence of bacteria in fetal tissues, we performed SEM on freshly isolated fetal intestine (n = 4 independent fetal gut samples), specifically the region around the ileum, shown to have the most diverse and rich microbiota within the intestinal tract (Dieterich et al., 2018; James et al., 2020; Rackaityte et al., 2020). The environmental contamination was minimized by performing sample isolation and processing under sterile conditions as discussed above (Figures S2A and S2B), and the dissected regions were fixed immediately with 2.5% glutaraldehyde. Interestingly, we observed bacteria-like morphological structures, resembling bacterial cocci, clustered together within limited regions across fetal gut at the 14^th^ weeks of gestation (n = 3) (Figure 4A). Such bacterial cocci were observed with an average size of 1–2 μm and were specifically nested within mucin-like structures present along the intestinal lumen, which were speckled across the ileum. Three of the four samples were from 14-weeks EGA fetuses and showed a similar bacterial localization pattern, whereas the fourth sample, belonging to a 10-weeks EGA fetus, did not show any significant bacterial localization across the ileum (Figure 4A). Most regions did not show any bacterial localization, and notably, these areas were also devoid of mucin-like structures (Figure 4A). Multiple scans across different regions showed similar localization patterns and morphologies in all three 14-weeks EGA fetuses, with discrete bacterial structures closely packed within meconium-associated mucin-like threads (Figure S4A). For one of the 14-weeks samples, we also scanned the upper (fore-gut) and lower (hind-gut) regions and observed similar bacterial entities albeit at a lower frequency compared to the ileum (Figure S4B). These observations revealed discrete clusters of bacterial structures present in fetal gut, consistent with the bacterial cocci-specific morphological embedding within meconium-associated mucin-like structures. We also performed SEM on the culture isolates from fetal gut and lung, wherein we observed distinct cocci-like bacteria, further suggesting that these fetal gut-associated bacteria are alive and can be propagated further (Figures 4B and S4C).

To further complement these morphological observations, we performed RNA-ISH using a eubacteria-specific 16S rRNA probe. Interestingly, we observed distinct localization of bacterial RNA, tightly packed alongside intestinal epithelium (Epcam^+^) within the fetal gut lumen (Figure 4C, H&E staining image of fetal gut cross-section in Figure S4D). Notably, these bacteria were not observed on the stromal side of the intestine and were specifically present within the luminal cavity. The bacterial rRNA signal was absent in corresponding fetal liver and mesenteric lymph node (mLN) samples (Figures S4E and S4F). Quantification of the bacterial signal (with respect to DAPI staining) showed significant enrichment of eubacterial 16S rRNA in fetal gut as compared to fetal liver and mLN (n = 3) (Figures 4D and 4E). Taken together, these observations demonstrate direct presence of bacteria within the fetal gut lumen during the 2^nd^ trimester of gestation.

### Fetal bacteria prime syngeneic T cells and induce expansion and memory activation of fetal T cells

**Figure 5:**
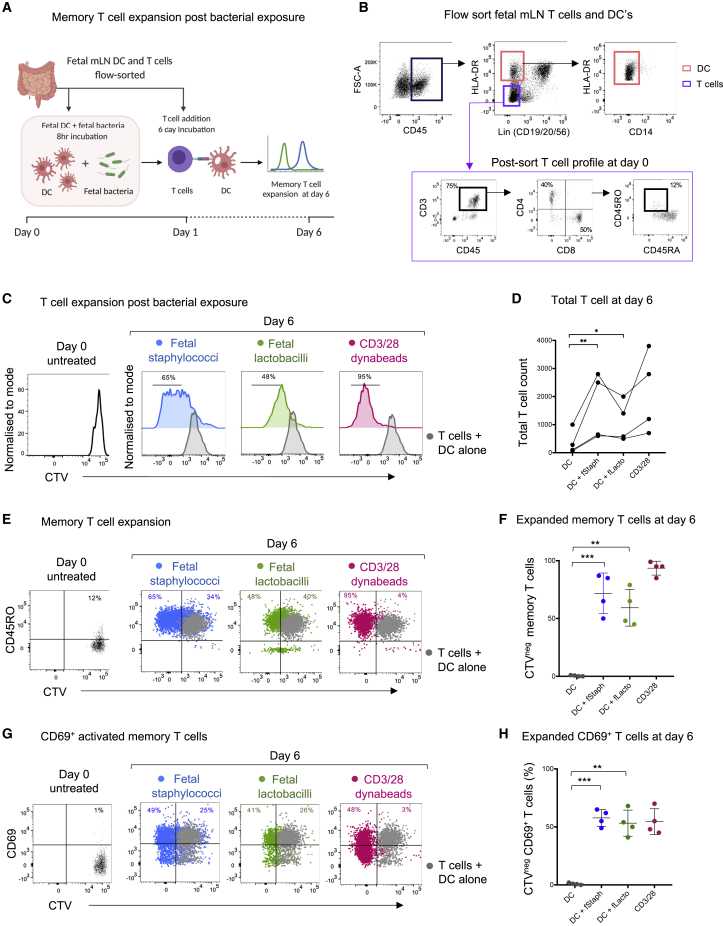
Syngeneic T cell expansion and memory activation assay for fetal T cells primed with fetal bacteria (A) Schematic representation of the syngeneic T cell expansion assay. DCs isolated from fetal mLN were primed with heat-killed fetal bacteria (for 8 h) and presented to CTV-labeled T cells from the same organ (mLN). At day 6, T cell expansion is assayed by dye dilution using flow cytometry. (B) Sort profile of fetal DCs and T cells for T cell expansion assay. HLA-DR high and lineage negative cells were selected for DC population, which were further enriched for CD14-negative cells. T cells were sorted by negative selection by gating on lineage-negative and HLA-DR-negative cells. Further, to assess the purity of T cells, a small portion was assayed by post-sort for T cell profile and found to be more than 75% pure. (C) Representative flow cytometry plots for T cell expansion (16 weeks EGA fetal mLN). CTV-labeled T cells were incubated with bacteria (fetal *Staphylococci* or *Lactobacilli*)-primed DCs for 6 days. For controls, T cells were incubated with unprimed DCs (no bacteria) or CD3/28 Dynabeads as a negative and positive control, respectively. T cell expansion is assayed by measuring dye dilution using flow cytometry. The conditions shown are T cells + DCs alone (gray), T cells + DCs primed with fetal *Staphylococci* (blue), T cells + DCs primed with fetal *Lactobacilli* (green), and T cells + CD3/28 Dynabeads (magenta). For comparisons, T cell profile at day 0 is shown at the left. (D) Total T cell number (absolute count) as counted by CountBright beads and manually, post 6 day incubation, plotted for all the conditions (n = 4). Statistical significance between experimental groups was determined by paired t test (^∗∗∗∗^p < 0.0001, ^∗∗∗^p < 0.001, ^∗∗^p < 0.01). (E) CD45RO^+^ memory T cell expansion post 6 day incubation measured by CD45RO expression and dye dilution (CTV^neg^) for all the conditions in comparison to T cell + DCs alone (gray). For comparisons, T cell at day zero is shown at the left. (F) Frequency of CTV^neg^ expanded memory T cells (CD45RO^+^, CTV^neg^) plotted for all conditions. (G) CD69^+^ activated memory T cell expansion post 6 day incubation measured by CD69 expression and dye dilution (CTV^neg^) for all the conditions, in comparison to T cell + DCs alone (gray). For comparisons, T cell at day 0 is shown at the left. (H) Frequency of expanded CD69^+^ active memory T cells (CTV^neg^) plotted for all conditions. Statistical significance between experimental groups was determined by two-tailed, unpaired Student’s t test (^∗∗∗∗^p < 0.0001, ^∗∗∗^p < 0.001, ^∗∗^p < 0.01). See also Figure S5.

**Figure S5:**
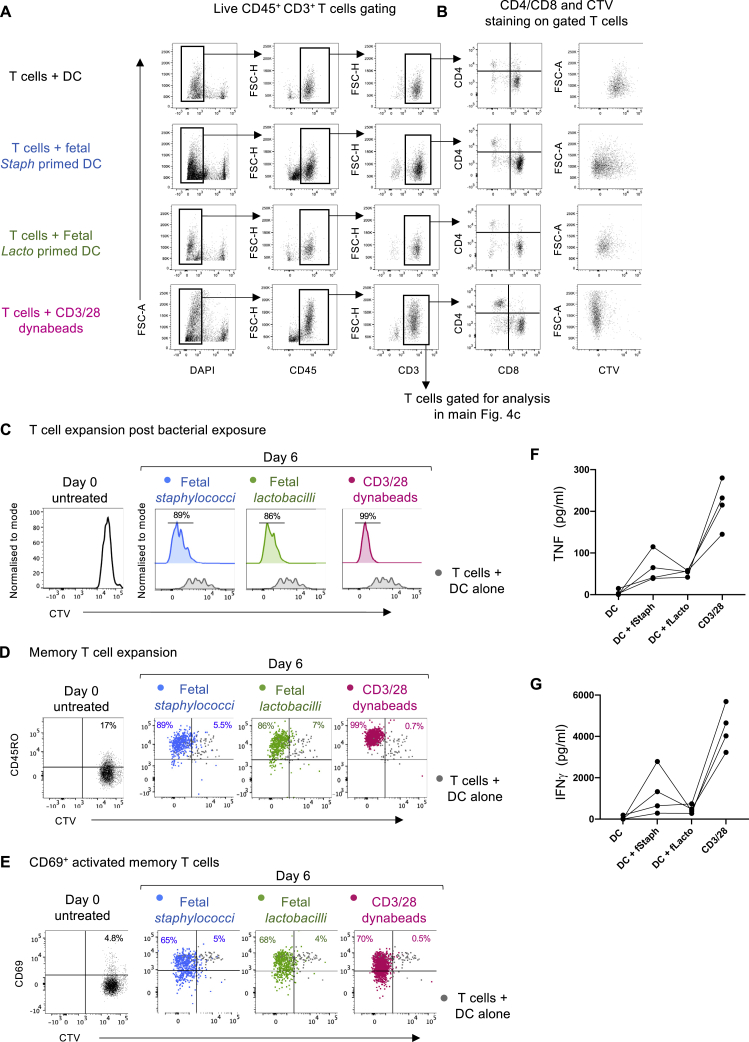
Gating strategy for *in vitro* syngeneic T cell expansion assay, related to Figure 5 (A) Reference input gating strategy for the Figure 5C, showing the live CD45^+^ CD3^+^ T cells gate obtained from fetal mesenteric lymph nodes T cells, post 6 days of incubation with the T cells + DC alone, T cells + DC primed with fetal *Staphylococcus*, T cells + DC primed with fetal *Lactobacillus* and T cells + CD3/28 dynabeads respectively (color coded in the figure). (B) As shown, the CD4/CD8 population frequencies post culturing, and CTV staining, gated on total CD3^+^ cells from the above panel a. (C) Another representative flow-cytometry plots for T cell expansion (15 weeks EGA fetal mLN). CellTrace violet (CTV) dye labeled T cells were incubated with bacteria (fetal *Staphylococci* and *Lactobacilli)* primed DC for 6 days. For controls, T cells were incubated with unprimed DC (no bacteria) or CD3/28 dynabeads as a negative and positive control, respectively. T cell expansion is assayed by measuring dye dilution using flow cytometry. The conditions shown are T cells + DC alone (gray), T cells + DC primed with fetal staphylococci (blue), T cells + DC primed with fetal lactobacilli (green) and T cells + CD3/28 dynabeads (magenta). For comparisons, T cell profile at day zero is shown at the left. (D) CD45RO^+^ memory T cell expansion post 6 day incubation measured by CD45RO expression and dye dilution (CTV^neg^) for all the conditions in comparison to T cell + DC alone (gray). For comparisons, T cell at day zero is shown at the left. (E) CD69^+^ activated memory T cell expansion post 6 day incubation measured by CD69 expression and dye dilution (CTV^neg^) for all the conditions, in comparison to T cell + DC alone (gray). For comparisons, T cell at day zero is shown at the left. (F) Levels of TNF released in the culture supernatant of T cells expanded post 6 day exposure to fetal bacteria as determined by drop array-based Luminex assays (n = 4) (G) Levels of IFNγ released in the culture supernatant of T cells expanded post 6 day exposure to fetal bacteria as determined by drop array-based Luminex assays (n = 4)

Our data demonstrate the presence of specific bacterial signals across fetal organs, specifically the fetal gut, although it is not clear what the physiological significance of these microbes could be. The effect of microbe-derived signals on the priming and maturation of the postnatal immune system in general is quite well established (Belkaid and Hand, 2014; Chung et al., 2012). Hence, we hypothesized that fetal microbial antigens could be one of the active forces behind the diverse T cell responses observed in fetal tissues. We thus proceeded to explore their potential cross-talk with the fetal immune system. Commensal microbes are known to elicit tissue-specific T cell memory responses, which get activated upon any potential breach in mucosal barrier (Belkaid and Tarbell, 2009; Belkaid et al., 2013; Hand et al., 2012; Harrison et al., 2019; Hegazy et al., 2017; Homann et al., 2001; Linehan et al., 2018; Schreurs et al., 2019; Sorini et al., 2018; Whibley et al., 2019). In the case of the gut, it is proposed that the microbe-specific memory T cells reside in the gut-associated mLN (Belkaid et al., 2013). In adult mice, microbial antigen-laden DCs are known to migrate to mLN thereby inducing microbe-specific T cell expansion (Belkaid et al., 2013; Hand et al., 2012; Leech et al., 2019; Linehan et al., 2018). We have previously shown that human fetal DCs migrate to the fetal mLN as early as the 16^th^ weeks of gestation (McGovern et al., 2017) and thus hypothesized that these migratory DCs may expose the T cells in fetal mLN to microbial antigens and prime them against microbial entities within fetal tissues. To test if fetal T cells could be recalled by DC presenting fetal bacteria-derived antigens, we recapitulated a minimalistic fetal environment *in vitro* with an autologous T cell expansion and activation assay (Figure 5A). We first sorted HLA-DR^+^ DCs and T cells from the same fetal mLN (Figure 5B). T cells were purified by negative selection to minimize activation, followed by labeling with a fluorescent dye CellTrace violet (CTV), prior to bacterial exposure. Sorted DCs were incubated with heat-killed fetal *Lactobacilli* or fetal *Staphylococci* for 8 h prior to coculture with T cells. These two bacteria were chosen because they were consistently present in fetal tissues in both high-throughput 16S rRNA gene sequencing and culture-based methods. Bacteria primed DCs were then presented to fetal T cells and their expansion was monitored post 6 days of incubation. For negative control, un-primed DCs (DCs alone, no bacteria) were incubated with T cells and CD3/28 Dynabeads were used for positive control. Fetal *Staphylococci* antigen-exposed T cells showed an average 70% (±15%) memory expansion, whereas the lactobacilli-exposed T cells showed ∼55% (±13%) memory expansion, in comparison to unprimed DC control (Figure 5C, input gating strategy and representative flow cytometry plots in Figures S5A–S5E). Upon exposure with fetal bacteria primed DCs, we observed a significant increase in total T cell count, demonstrating the expansion of T cells post bacterial priming (Figure 5D). Notably, the magnitude of T cell expansion with *Staphylococci*-primed DCs was consistently higher than with *Lactobacilli*, suggesting a bacteria specific heterogeneity in fetal T cell responses. Positive control (CD3/28 Dynabeads) showed more than ∼95% expansion throughout. Assessment of TNF and IFNγ production post 6 day bacterial exposure in culture supernatants revealed that both TNF and IFNγ were abundantly produced and released upon fetal *Staphylococci* exposure (Figures S5F and S5G). Interestingly, we observed a significant increase in CD45RO expression post expansion, indicating a potential memory phenotype against microbial antigens, whereas only <12% of memory T cells (CD45RO^+^) were present at day 0 prior to exposure (Figures 5E and 5F). Interestingly, there was a significant increase in the activation and tissue residency marker CD69 (∼50% versus 1% on day 0) post-expansion, suggesting the early stages of memory activation upon bacterial exposure (Figures 5G and 5H).

**Figure 6:**
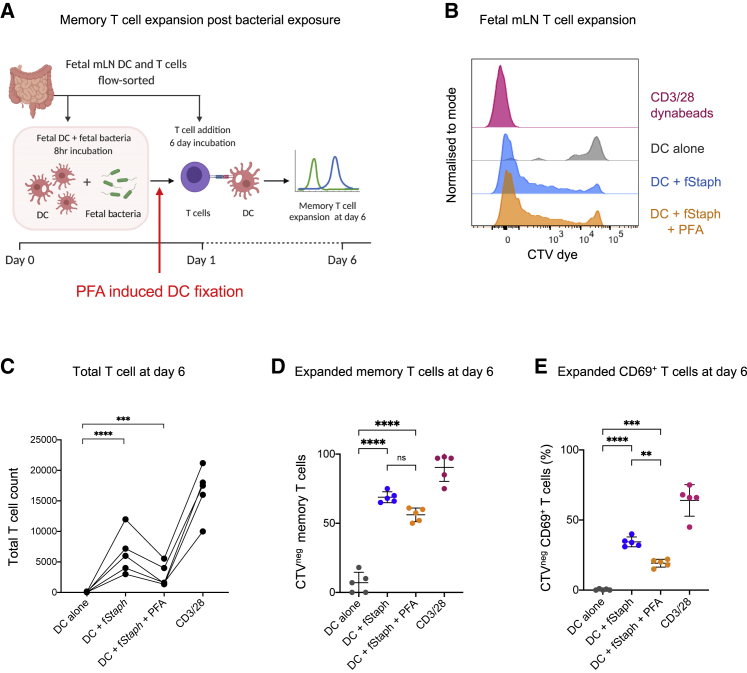
Fetal T cell expansion and memory activation with PFA-fixed DCs (A) Schematic representation of the syngeneic T cell expansion assay. DCs isolated from fetal mLN were primed with heat-killed fetal bacteria (for 8 h) followed by 1% PFA-induced fixation. Fixed DCs were then presented to CTV-labeled T cells from the same organ (mLN). At day 6, T cell expansion is assayed by dye dilution using flow cytometry. (B) Representative flow cytometry histograms for T cell expansion (17-weeks EGA fetal mLN). CTV-labeled T cells were incubated with bacteria (fetal *Staphylococci*) primed DCs for 6 days. For controls, T cells were incubated with unprimed DCs (no bacteria) or CD3/28 Dynabeads as a negative and positive control, respectively. T cell expansion is assayed by measuring dye dilution using flow cytometry. The conditions shown are T cells + DCs alone (gray), T cells + fetal *Staphylococci* primed DCs (blue), T cells + fetal *Staphylococci* primed PFA-fixed DCs (orange), and T cells + CD3/28 Dynabeads (magenta). (C) Total T cell number (absolute count) as counted by CountBright beads, post 6 day incubation, plotted for all the conditions (n = 4). Statistical significance between experimental groups was determined by paired t test (^∗∗∗∗^p < 0.0001, ^∗∗∗^p < 0.001, ^∗∗^p < 0.01). (D) Frequency of CTV^neg^-expanded memory T cells (CD45RO^**+**^, CTV^neg^) plotted for all conditions. (E) Frequency of expanded CD69^+^ active memory T cells (CTV^neg^) plotted for all conditions. Statistical significance between experimental groups was determined by two-tailed, unpaired Student’s t test (^∗∗∗∗^p < 0.0001, ^∗∗∗^p < 0.001, ^∗∗^p < 0.01). See also Figure S6.

**Figure S6:**
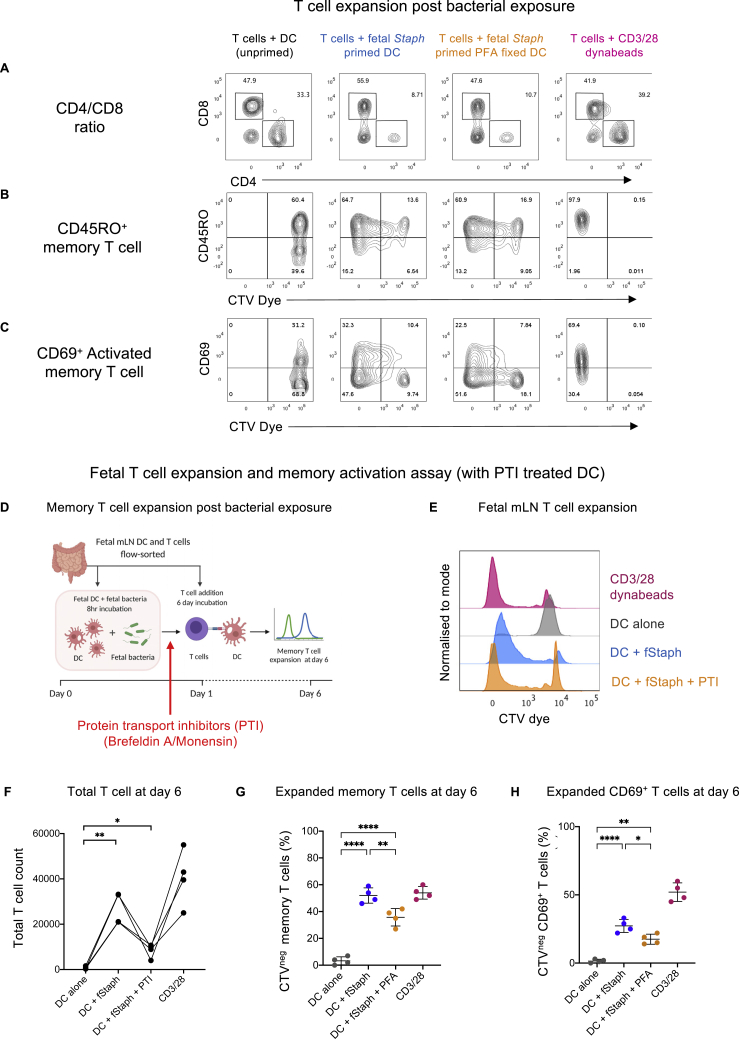
Flow-cytometry plots for *in vitro* syngeneic T cell expansion assay (with PFA fixed DC), related to Figure 6 Representative flow cytometry plots of *in vitro* syngeneic T cell expansion of fetal mLN T cells (treated with bacteria primed DC fixed with 1% PFA) showing the: (A) frequency of CD4 and CD8 T cells ratio, (B) CD45RO^+^ memory T cell plotted against CTV dye to show the dye dilution post 6 days, and (C) CD69^+^ activated memory T cell plotted against CTV dye to show the dye dilution post 6 days. The conditions shown are T cells + fetal staphylococci primed DC (blue), T cells + fetal staphylococci primed PFA fixed DC (orange), T cells + unprimed DC alone (gray), and T cells + CD3/28 dynabeads (magenta). Fetal T cell expansion and memory activation assay (with PTI treated DC) (D) Schematic representation of the syngeneic T cell expansion assay. DC isolated from fetal mLN were primed with heat-killed fetal bacteria (for 8 hours) followed by Protein Transport Inhibitor (PTI) treatment (a combination of Monensin and Brefeldin A). PTI treated DC were then presented to CTV labeled T cells from the same organ (mLN). At day 6, T cell expansion is assayed by dye dilution using flow cytometry. (E) Representative flow-cytometry histograms for T cell expansion (17 weeks EGA fetal mLN). CellTrace Violet (CTV) dye labeled T cells were incubated with bacteria (fetal staphylococci) primed DC for 6 days. For controls, T cells were incubated with unprimed DC (no bacteria) or CD3/28 dynabeads as a negative and positive control, respectively. T cell expansion is assayed by measuring dye dilution using flow cytometry. The conditions shown are T cells + DC alone (gray), T cells + fetal staphylococci primed DC (blue), T cells + fetal staphylococci primed PTI treated DC (orange) and T cells + CD3/28 dynabeads (magenta). (F) Total T cell number (absolute count) as counted by CountBright beads, post 6 day incubation, plotted for all the conditions (n = 4). Statistical significance between experimental groups was determined by paired t test (^∗∗∗∗^p < 0.0001, ^∗∗∗^p < 0.001, ^∗∗^p < 0.01) (G) Frequency of CTV^neg^ expanded memory T cells (CD45RO^**+**^, CTV^neg^) plotted for all conditions. (H) Frequency of expanded CD69^+^ active memory T cells (CTV^neg^) plotted for all conditions. Statistical significance between experimental groups was determined by two-tailed, unpaired Student’s t test (^∗∗∗∗^p < 0.0001, ^∗∗∗^p < 0.001, ^∗∗^p < 0.01)

Next, we aimed to investigate if the effect on T cell activation observed post bacterial exposure is specific to antigen presentation. We fixed fetal DCs post bacterial priming with 1% paraformaldehyde (PFA), prior to T cell presentation, to ensure that the responses obtained reflected only the processing events that occurred due to antigen exposure (Figure 6A). We selected fetal isolated *Staphylococci* for this priming because it was specifically enriched in the fetal gut (Figures 2G and 2H) and showed significant T cell activation in the previous assay (Figure 5). Interestingly, we observed similar fetal T cell expansion profile with both fixed and non-fixed DCs; however, the extent of proliferation was lower in fixed DCs as measured by absolute T cell count post 6 days of exposure (Figures 6B and 6C). The number of T cells surviving and proliferating with live DCs was higher than fixed DCs, although both were significantly higher than the un-primed DC control (Figure 6C). This observation suggests the effect of bacterial exposure on fetal T cell expansion is contributed by both antigen presentation as well as cytokine-mediated signals. However, antigen presentation alone is sufficient to drive the proliferation of a significant number of T cells in response to bacterial signals. These expanded T cells were CD45RO^+^ and a significant proportion was CD69^+^, suggestive of an activated memory response toward fetal bacteria (Figures 6D and 6E, representative flow cytometry plots in Figures S6A–S6C). To further validate these observations with an independent method, we also used protein transport inhibitors (PTI) (combination of monensin and brefeldin A) to block cytokine production post bacterial priming of DCs (Figure S6D). This approach also showed similar T cell expansion and activation response as in the case of PFA-induced fixation (Figures S6E–S6H).

Taken together, these observations suggest that fetal mLN T cells exhibit an activated memory response toward microbial antigens encountered *in utero*. Further studies are required to explore the antigenic specificities of these T cell-microbial interactions in human fetal organs and the synergy with a non-antigen specific (cytokine-mediated) response toward the extent of proliferation.

## Discussion

The events occurring during fetal gestation are essential for the overall development and growth of the individual. Key factors transferred from the mother to the fetus during this process establish the cornerstones of early life immunity and life-long tolerance. Various studies have recently suggested that certain antigens as well as bacterial entities may cross the placental barrier and make their way to fetal organs (Aagaard et al., 2014; Cao et al., 2014; Li et al., 2019; McGovern et al., 2017; Mold et al., 2008; Rackaityte et al., 2020; Schreurs et al., 2019; Stras et al., 2019; Willyard, 2018). A recent report from our group also demonstrated that human fetal mast cells are primed and sensitized during gestation through maternal immunoglobulin E (IgE) that cross the placental barrier (Msallam et al., 2020). Interestingly, a recent report on plant development showed partial inheritance of plant microbiome by vertical transmission through seedling (Abdelfattah et al., 2021). All these observations open a new avenue in developmental immune-priming wherein maternal-derived microbial entities may condition the progeny toward future antigenic exposures in life.

Our findings demonstrate that healthy human fetal tissues (in the 2^nd^ trimester of gestation) contain effector Tems, a sparse biomass of bacteria, and an active memory T cell response toward fetal bacteria. We also demonstrate direct spatial localization of bacterial entities, localized within the lumen of developing fetal gut, during the 2^nd^ trimester of gestation. These data are in line with recent studies that indicated antigenic priming of the fetal immune system begins during gestation (Halkias et al., 2019; Li et al., 2019; McGovern et al., 2017; Rackaityte and Halkias, 2020; Rackaityte et al., 2020; Schreurs et al., 2019; Stras et al., 2019). However, our study demonstrates for the first time that such microbial presence primes the fetal immune system, thereby putting early microbial memory in the context of fetal immune priming, a concept not explored before in fetal immunity. It will be interesting to explore the precise nature of these microbial antigen-specific circulatory and tissue-resident immune cells in human fetal organs, and their potential role in imparting selective defense against pathogenic microbes in neonatal and adult life. Taken together, these findings have wider implications in understanding the key factors involved in fetal immune system development and priming *in utero*, which may set the basis for life-long health and immunity of the organism.

Although we have taken particular care to minimize the risk of contaminations in this study, we cannot completely exclude potential confounders due to environmental factors. In particular, fetal skin and placenta samples will have been exposed to vaginal microbiota during delivery or by the operator’s skin (Chen et al., 2017; Ravel et al., 2011). However, the existence of a spatially diverse microbial signal in fetal tissues, their ability to culture-expand anaerobically, and the presence of microbial antigen-specific memory T cell activation, are difficult to reconcile with either systematic biases or random noise. Collectively, our data suggest a low but consistent presence of microbes in at least some of the healthy human fetuses in the 2^nd^ trimester of gestation. Recent reports of the presence of microbes in fetal lung and gut provide independent assertions to our findings (Al Alam et al., 2020; Rackaityte et al., 2020). However, this is an area of constant exploration and evidence from various spheres of technological advances is needed to even partially understand the dynamics of fetal development and immune priming.

### Limitations of the study

This study provides multiple evidence for the presence of live microbes in human fetal tissues, however, there are several outstanding limitations to be addressed in future studies. First, the precise source of bacteria in fetal tissues still remains unknown. More clinical studies with access to pertinent maternal samples are required to determine the source of origin and the trajectory of bacterial entry to fetal organs. Second, although we have undertaken a wide array of controls in this study, contamination from maternal organs, especially from the lower genital tract at delivery, cannot be formally ruled out. Sequencing the maternal uterine microbiome, possibly at the time of delivery, and the fetal microbes may address this issue. We acknowledge that the species/genera specificities for fetal organs is limited in this study, and more detailed metagenomics approaches will be required to precisely determine the key bacterial species present in fetal tissues and their niche preferences.

Last, the antigenic specificities of fetal T cell-microbial interactions and its synergy with a non-specific antigen response (cytokine-mediated) needs further exploration as we cannot formally rule out the possible contribution of a super-antigen-based activation or a pan-bacterial response against fetal bacteria. Further studies are required to address these possibilities in the future.

## STAR★Methods

### Key resources table

**Table:** 

REAGENT or RESOURCE	SOURCE	IDENTIFIER
**Antibodies (flow cytometry)**		

CD20 (clone 2H7) FITC	BioLegend	Cat# 302304, RRID: AB_314252
CD56/NCAM (clone MEM-188) FITC	BioLegend	Cat# 304604, RRID: AB_314446
CD19 (clone HIB19) FITC	BioLegend	Cat# 302206, RRID: AB_314236
CD45 (clone HI30) V500	BD Biosciences	Cat# 560777, RRID: AB_1937324
CD14 (clone M5E2) Alexa Fluor 700	BD Biosciences	Cat# 557923, RRID: AB_396944
HLA-DR (clone LN3) PerCP-Cyanine5.5	Thermo Fisher Scientific	Cat# 45-9956-42, RRID: AB_10718537
CD45RA (clone HI100) BV605	BD Biosciences	Cat# 562886, RRID: AB_2737865
CD4 (clone OKT4) APC	BioLegend	Cat# 317416, RRID: AB_571945
CD152/CTLA-4 (clone BNI3) PerCP/Cyanine5.5	BioLegend	Cat# 369608, RRID: AB_2629674
CD25 (clone BC96) PE/Dazzle 594	BioLegend	Cat# 302646, RRID: AB_2734260
CD8A (clone RPA-T8) PE	BioLegend	Cat# 301008, RRID: AB_314126
CD3 (clone UCHT1) BUV395	BD Biosciences	Cat# 563546, RRID: AB_2744387
CD45RO (clone UCHL1) PE-Cy7	BD Biosciences	Cat# 337168, RRID: AB_647426
CD69 (clone FN50) Biotin	BioLegend	Cat# 310924, RRID: AB_2074958
Streptavidin BUV737	BD Biosciences	Cat# 612775, RRID: AB_2870104
DAPI Solution	Thermo Fisher Scientific	Cat# 62248

**Antibodies (CyTOF)**		

CD3 (clone UCHT1) metal channel 139	BioLegend	Cat# 300402, RRID: AB_314056
CD4 (clone SK3) metal channel 148	BioLegend	Cat# 344625, RRID: AB_2563749
CD8 (clone SK1) metal channel 144	BioLegend	Cat# 344727, RRID: AB_2563762
CD11b (clone ICRF44) metal channel 209	Fluidigm	Cat# 3209003, RRID: AB_2687654
CD14 (clone M5E2) metal channel 112/114	BioLegend	Cat# 301802, RRID: AB_314184
IL-4 (clone 8D4-8) metal channel 145	BioLegend	Cat# 500707, RRID: AB_315119
GATA3 (clone TWAJ) metal channel 151	Thermo Fisher Scientific / eBioscience	Cat# 14-9966-82, RRID: AB_1210519
IFN-γ (clone B27) metal channel 168	BioLegend	Cat# 506513, RRID: AB_315446
T-bet (clone 4B10) metal channel 163	Bio X Cell	Cat# BE0100, RRID: AB_10950173
IL-17A (clone BL168) metal channel 169	BioLegend	Cat# 512302, RRID: AB_961399
ROR gamma (t) (clone AFKJS-9) metal channel 162	Thermo Fisher Scientific / eBioscience	Cat# 14-6988-82, RRID: AB_1834475
CD45RA (clone HI100) metal channel 171	BioLegend	Cat# 304102, RRID: AB_314406
CD45RO (clone UCHL1) metal channel 142	BioLegend	Cat# 304202, RRID: AB_314418
CD69 (clone FN50) metal channel 167	BioLegend	Cat# 310902, RRID: AB_314837
CD152 (clone BNI3) metal channel 155	BD Biosciences	Cat# 555851, RRID: AB_396174
CD274/PD-L1 (clone 29E.2A3) metal channel 156	BioLegend	Cat# 329719, RRID: AB_2565429
HLA-DR (clone L243) metal channel 143	BioLegend	Cat# 307612, RRID: AB_314690
LAG3 (clone 17B4) metal channel 170	Abcam	Cat# ab40466, RRID: AB_776098
CD279/PD-1 (clone EH12.2H7) metal channel 147	BioLegend	Cat# 329941, RRID: AB_2563734
Ki-67 (clone 20Raj1) metal channel 166	Thermo Fisher Scientific / eBioscience	Cat# 14-5699-82, RRID: AB_2016711
CD31 (clone WM59) metal channel 172	BioLegend	Cat# 303102, RRID: AB_314328
TCR-gamma delta - FITC (clone 5A6.E9)	Thermo Fisher Scientific	Cat# TCR1061, RRID: AB_223500
Anti-FITC (clone FIT-22) metal channel 146	BioLegend	Cat# 408302, RRID: AB_528901
TCR-αβ-PE (clone T10B9.1A-31)	BD Biosciences	Cat# 555548, RRID: AB_395932
Anti-PE (clone PE001) metal channel 176	BioLegend	Cat# 408102, RRID: AB_2168924
CD197/CCR7 (clone G043H7) metal channel 154	BioLegend	Cat# 353202, RRID: AB_10945157
CXCR5 (clone RF8B2) metal channel 159	BD Biosciences	Cat# 552032, RRID: AB_394324
CD25 (clone M-A251) metal channel 150	BD Biosciences	Cat# 555429, RRID: AB_395823
CD127 (clone A019D5) metal channel 149	BioLegend	Cat# 351302, RRID: AB_10718513
FOXP3 (clone PCH101) metal channel 165	Thermo Fisher Scientific / eBioscience	Cat# 14-4776-82, RRID: AB_467554
CD304 (clone 3E12) metal channel 157	BioLegend	Cat# 145202, RRID: AB_2561841
Helios (clone 22F6) metal channel 174	BioLegend	Cat# 137202, RRID: AB_10900638
TGF-β1 (LAP) (clone TW4-2F8) metal channel 153	BioLegend	Cat# 349602, RRID: AB_10645476
IL-10 (clone JES3-9D7) metal channel 158	BioLegend	Cat# 501402, RRID: AB_315168
TNFα (clone MAb11) metal channel 152	BioLegend	Cat# 502902, RRID: AB_315254
Granzyme B (clone CLB-GB11) metal channel 173	Abcam	Cat# ab103159, RRID: AB_10715242
IL-2 (clone MQ1-17H12) metal channel 164	BioLegend	Cat# 500302, RRID: AB_315089
IL-15 (clone BH1509) metal channel 160	BioLegend	Cat# 515002, RRID: AB_2561319
GM-CSF (clone BVD2-21C11) metal channel 161	BioLegend	Cat# 502302, RRID: AB_315216
CD45-A (clone HI30) metal channel 89	Fluidigm	Cat# 3089003, RRID: AB_2661851
CD45-B, C or D (clone HI30) metal channel 115, 141, 175	BioLegend	Cat# 304002, RRID: AB_314390
Cell-ID Intercalator-Ir metal channel 191/193	Fluidigm	Cat# 201192B
Cisplatin (Live/Dead) metal channel 195	Sigma-Aldrich	Cat# 15663-27-1

**Biological samples**		

Adult blood samples	National University Hospital (NUH)	N/A
Human adult Gut and Lung samples	National University Hospital (NUH)	N/A
Human fetal samples, fetal blood, and cord blood	Division of Obstetrics and Gynaecology, KK Women’s and Children’s Hospital, Singapore	N/A
Formalin-fixed, paraffin-embedded (FFPE) blocks of fetal tissues	The Advanced Molecular Pathology Laboratory (AMPL) of Institute of Molecular and Cell Biology (IMCB)	N/A
H&E staining and paraffin unstained slides	The Advanced Molecular Pathology Laboratory (AMPL) of Institute of Molecular and Cell Biology (IMCB)	N/A

**Critical commercial assays**		

HyClone RPMI 1640 Media	Cytiva (Formerly GE Healthcare Life Sciences)	Cat# SH3025501
Iscove’s Modified Dulbecco’s Medium (1x)	Thermo Fisher Scientific	Cat# 12440053
Human Serum	Sigma-Aldrich	Cat# H4522
CellTrace Violet Cell Proliferation Kit	Thermo Fisher Scientific	Cat# C34557
Human IL-2 IS premium grade	Miltenyi Biotec	Cat# 130-097-745
Human Flt3-Ligand premium grade	Miltenyi Biotec	Cat# 130-096-479
Dynabeads Human T-Activator CD3/CD28	Thermo Fisher Scientific	Cat# 11131D
Sodium Pyruvate (100mM)	GIBCO	Cat# 11360-070
Penicillin-Streptomycin	GIBCO	Cat# 15140122
LB Broth Miller, Bacterial Culture Media	1^st^ BASE	Cat# BIO-4000-1kg
Cyclopore Polycarbonate Membrane	Whatman	Cat# 7060-2501
Stainless Steel Insect Pin	Roboz	Cat# RS-6082-25
Osmium Tetroxide, 1 g crystal	Ted Pella, Inc.	Cat# 18456
Glutaraldehyde, 25% EM grade, 100ml	Ted Pella, Inc.	Cat# 18427
RNAscope Multiplex Fluorescent Reagent Kit v2 Assay	ACDBio	Cat# 323100
RNAscope Probe Diluent	ACDBio	Cat# 300041
Opal 7-Color kit	Perkin Elmer	Cat# NEL821001KT
Xylenes	Sigma	Cat# 534056
RNAscope Probe- EB-16S-rRNA	ACDBio	Cat# 464461
RNAscope Probe - Hs-EPCAM	ACDBio	Cat# 310281-C3
ProLong Diamond AntifadeMountant	Thermo Fisher Scientific	Cat# P36970

**Deposited data**		

Raw data files for 16S rRNA sequencing	NCBI Gene Expression Omnibus	GEO: GSE169306

**Software and algorithms**		

inForm® Tissue Analysis Software	Akoya Biosciences	https://www.akoyabio.com/phenoptics/software/inform-tissue-finder/
Phenochart Whole Slide Viewer	Akoya Biosciences	https://www.akoyabio.com/support/software/
ImageJ	National Institutes of Health and the Laboratory for Optical and Computational Instrumentation	https://imagej.nih.gov/ij/
Cell Profiler	Broad’s Institute of Imaging Platform	https://cellprofiler.org/
Diva	BD Biosciences	https://www.bdbiosciences.com/en-us
FlowJo v.10.5.3	Tree Star Inc.	https://www.flowjo.com
GraphPad Prism version 7 and 9	Graphpad	https://www.graphpad.com/scientific-software/prism/
R 4.4	The R Foundation	https://www.r-project.org
UMAP	(McInnes et al., 2018)	https://github.com/lmcinnes/umap
SMuRF 1.0	CRAN	https://cran.r-project.org/web/packages/smurf/index.html
QIIME 1.8.0	QIIME	http://qiime.org/
emperor 1.0	Biocore	https://biocore.github.io/emperor/
vegan v2.5.7	CRAN	https://cran.r-project.org/web/packages/vegan/index.html
Python 2.7.0	Python Software Foundation	https://www.python.org/
Python 3.7.0	Python Software Foundation	https://www.python.org/
matplotlib 3.2.1	PyPI	https://pypi.org/
numpy 1.19.4	PyPI	https://pypi.org/
pandas 0.25.3	PyPI	https://pypi.org/
seaborn 0.9.0	PyPI	https://pypi.org/

### Resource availability

#### Lead contact

Further information and requests for resources and reagents should be directed to and will be fulfilled by the Lead Contact:

Florent Ginhoux (florent_ginhoux@immunol.a-star.edu.sg)

#### Materials availability

This study did not generate new unique reagents.

#### Data and code availability

All the raw sequencing data reported in this paper is available on GEO and the accession number is GSE169306 (Human fetal immunity and microbial priming during early development). The authors declare that all data supporting the findings of this study are available within the manuscript and/or its Supplementary Files or available on request.

### Experimental model and subject details

#### Human samples

Human fetal and adult tissues, cord blood, adult blood and amniotic fluid, were obtained in accordance from Singapore SingHealth and National Health Care Group Research Ethics Committees. All women gave written consent to the use of fetal tissues according to internationally recognized guidelines (Polkinghorne, 1989). Review of the guidance on the research use of fetuses and fetal material. CM 762). All fetal tissues (placenta, lung, thymus, spleen, gut, mesenteric lymph nodes, and skin) were obtained from 2nd trimester (12-22 weeks EGA) elective pregnancy terminations carried out for socio-psychological reasons. All fetuses were considered structurally normal on ultrasound examination prior to termination and by gross morphological examination following termination. Fetal tissues from 2^nd^ trimester of gestation were used for this study (Table S1). The study of fetal blood was approved by the Hôpital Erasme ethics committee (ULB). All participants (or mothers, in the case of fetal samples) gave written informed consent. We obtained fetal blood (between 23 and 37 weeks EGA) as described previously (Dimova et al., 2015).

#### Fetal dissection and organ collection

Mid-trimester terminations were medically induced by the use of prostaglandins, and the fetus was delivered through the birth canal. Fetal organs were collected under sterile conditions in a tissue culture hood. Aseptic equipment was used for collecting fetal tissues. Fetal skin was collected first. Due to the risk of contamination from vaginal microbes, great care was taken to minimize the risk of contamination of internal organs. Fetal skin across the abdomen was cut and pinned back to prevent it from touching internal organs. Fresh aseptic instruments were then used to remove internal organs. Fetal lungs were then removed, followed by the thymus, spleen, liver and gastrointestinal tract. The mesenteric lymph nodes were removed with the gastrointestinal tract. If a placenta was obtained, it was processed last. Tissues were processed as described below for suspension mass cytometry assays and microbiome studies. When tissues were used for both arms of the study, samples were divided using aseptic equipment. Sterile equipment was rinsed in PBS and this PBS was used as an environmental control, along with unused PBS and environment air swabs.

Fetal organ dissection in detail:

Prior to start of the harvest procedure, the entire bio-safety cabinet was thoroughly disinfected.

Order of Harvest- Skin, Liver, Gut, Spleen, Thymus, Lungs and Placenta

Separate (new and sterilized) dissection tools were used for dissection of each organ to avoid instrument associated contaminations as well as cross-tissue contaminations.

Skin: Starting the dissection of skin, using a scissor, an incision was made into the skin (back, chest and abdomen) and carefully separated the skin from the muscle, followed by isolation and transfer to buffer/media.
Liver: Abdominal cavity was entered, and the liver carefully detached from the diaphragm and the anterior abdominal wall, and placed in sterile buffer/media.
Intestines: The omentum and mesenteric was dissected, and intestines snipped near the rectum and at the gastric-duodenal junction, rinsed with PBS transferred to sterile buffer/media.
Spleen: The spleen was dissected from its splenic portal attachments, placed in sterile buffer/media.
Thymus: A cut is made along the sternum to the neck to access the thoracic cavity. The thymus can be identified as fatty tissue attached to the upper mediastinum, where it is isolated with a pair of sterile forceps, and placed in sterile buffer/media.
Lungs: Using the pointed/fine-tip forceps, a pinch on the bronchus was exerted and a scalpel was used to remove the lung from the thoracic cavity and transferred to a sterile buffer/media.
Placenta: one cm^3^ of placental tissues was obtained near the cord insertion and transferred to sterile buffer/media. This biopsy includes both fetal and decidual surfaces of the placenta.

#### Cell isolation for suspension mass cytometry assays

Fetal organs were mechanically dispersed and incubated with 0.2 mg/ml collagenase (TypeIV; Sigma Aldrich) and DNase I (20,000U/ml, Roche) in RPMI with 10% FCS for 1 hour in a 6 well plate. Viability was typically 80%–90% measured by DAPI exclusion (Partec). Fetal gut was initially cut longitudinally through the center, washed extensively in PBS until all inner content (meconium) was removed and the PBS was clear, before mechanical dispersion and digestion as above, for 1h. Adult lung specimens were obtained from peritumoral tissue. Adult lung specimens were prepared as described previously (Aiuti et al., 2013; Mattar et al., 2012). T cells were enriched isolated to 90% purity from adult and fetal organs by negative selection using T cell enrichment kits (Miltenyi Biotec) and separated on an AutoMacs following manufacturer’s instructions.

#### Suspension mass cytometry staining, **barcoding, acquisition and data** pre-processing

T cells were stained as previously described and then analyzed by CyTOF. Briefly, T cells were stimulated with phorbol 12-myristate 13-acetate (PMA) (150 ng/ml, Sigma-Aldrich) and ionomycin (750 ng/ml, Sigma-Aldrich) for 5h, blocked with secretory inhibitors, brefeldin A (1:1000, eBioscience) and monesin (1:1000, Biolegend) after one hour, for the last 4 hours, in 10% v/v human serum, 1% v/v PSG, RPMI at 37°C, 5% CO2. The cells were then washed and stained with cell viability dye cisplatin (200 μM, Sigma-Aldrich) for 5 min at room temperature. The cells were re-washed and each individual sample was barcoded with a unique combination of anti-CD45 conjugated with either heavy metal 89, 115, 141 or 175, as previously described for 25 min on ice (Lai et al., 2015; McGovern et al., 2017). The barcoded cells were washed and stained with a surface antibody cocktail in 4% v/v heat-inactivated FBS, 2mM EDTA, 0.05% w/v sodium azide in pH 7.4 PBS for 30 min on ice. The cells were again washed and re-suspended in fixation/permeabilization buffer (1:3, eBioscience) for 45 min on ice. The permeabilized cells were then stained with an intra-antibody cocktail (1:10, permeabilisation buffer, eBioscience) for 45 min on ice, before washing and staining with a DNA intercalator Ir-191/193 (1:2000 in 1.6% w/v paraformaldehye, Fluidigm) overnight at 4°C or for 20 min on ice. Finally, the cells were washed and re-suspended with EQTM Four Element Calibration beads (1:10, Fluidigm) in ultra-pure distilled water at 1x10^6^ cells/ml. The cell mixture was then loaded and acquired on a Helios mass cytometer (Fluidigm) calibrated with CyTOF Tunning solution (Fluidigm). The output FCS files were randomized and normalized with the EQTM Four Element Calibration beads against the entire run, as per the manufacturer’s recommendations. Key Resources Table contains all the antibodies with specifications used in CyTOF experiments.

#### UMAP Analysis

The automated analysis was performed by the UMAP algorithm as described previously (Becht et al., 2018). FlowJo software and R package were used for data analysis.

#### DNA extraction

DNA from fetal tissues was extracted according to the protocol as previously described (Rancati et al., 2008), except that the volume of Breaking Buffer and phenol/chloroform/IAA used was 400 μL. Fetal microbiome reagent and environmental controls were concurrently performed during the DNA extraction process (Table S2 contains all the primers used, if not already mentioned in the methods).

#### BactQuant qPCR

The qPCR was performed according to the protocol as previously described (Liu et al., 2012), with slight modifications. Briefly, the PCR reactions were run in 10uL reactions and were composed of the universal 16S BactQuant primers (5′-CCTACGGGDGGCWGCA-3′ and 5′-GGACTACHVGGGTMTCTAATC-3′) at 1.8uM, probe (6FAM-5′-CAGCAGCCGCGGTA-3′-MGBNFQ) at 225 nM, TaqMan Universal PCR MasterMix x2, water and undiluted sample DNA (3uL). The amplification was performed on a 7900HT Real Time PCR System (Applied Biosystems) with cycling conditions of 50°C for 3 min, 95°C for 2 min, and 40 cycles of 95°C for 15 s and 60°C for 1min. Cycle threshold value (Ct value) for each 16 S qPCR reaction were obtained using a manual Ct threshold of 0.02 and manual baseline between cycles 1-15 in the Sequence Detection Systems v2.4 software (Applied Biosystems). The qPCR for each sample was run as technical duplicates or more depending on sample availability, and the values were averaged to give the final Ct value used in data analysis.

#### High-throughput 16S rRNA gene sequencing at SIgN (Singapore Immunology Network)

Genomic DNA isolated from fetal tissues and control samples were treated with McrBC restriction enzyme (New England BioLabs) according to manufacturer’s instructions, to remove host DNA and to enrich for microbial DNA. The treated DNA samples were purified using 0.8X ratio of AMPure XP beads (Beckman Coulter). The McrBC-treated, purified gDNA was quantified using Quant-iT TM Picogreen® dsDNA Assay kit (Invitrogen). For amplification of the 16S rRNA gene-variable regions three rounds of amplification were performed using 100ng of McrBC-treated gDNA with LongAmp Taq DNA polymerase (New England Biolabs) according to manufacturer’s instructions. The first step aimed at enriching microbial sequences was performed using primers that amplify V2-V6 regions (Table S2). PCR cycling parameters consisted of initial denaturation step for 30 s at 94°C, followed by 12 cycles of 15 s at 94°C, 30 s at 45°C, and 1min at 65°C with a final extension for 10 min at 65°C. Subsequently a nested PCR was performed targeting V4-V5 regions using the enriched V2-V6 amplicon as template. PCR Cycling parameters for amplifying V4-V5 regions were similar to V2-V6 PCR cycling profile, except that the extension was for 30 s at 65°C. The final amplification step involved addition of barcodes for identifying, de-multiplexing individual samples and Illumina adaptor sequences to the V4-V5 enriched product using communal primers. Communal PCR amplification consisted of initial denaturation step for 30 s at 94°C, followed by 30 cycles of 15 s at 94°C, 30 s at 45°C, and 1 min at 65°C with a final extension for 10 min at 65°C. Equimolar concentrations of the prepared libraries were pooled and electrophoresed using 1% agarose gel. Pooled amplicon libraries were gel-purified using the Qiaquick Gel Extraction Kit (QIAGEN). Integrity of gel-purified libraries were validated using the High Sensitivity DNA kit (Agilent Technologies). The libraries were quantified using KAPA Library Quantification Kit (Kapa Biosystems) to ascertain the loading concentration and sequenced on a HiSeq 2500 System (Illumina) operated in Rapid Run Mode to generate 2 × 250 bp paired-end reads.

#### High-throughput 16S rRNA gene sequencing at WIS (Weizmann Institute of Science)

##### DNA Extraction

Samples from Fetus ID W1 to W10 (10 fetuses- Table S1) were processed at WIS. DNA from fetal tissues was isolated according to the protocol as previously described, designed to extract bacterial DNA (Geller et al., 2017; Nejman et al., 2020). Frozen tissues were extracted with the UltraClean Tissue & Cells DNA Isolation kit (MoBio - #12334) according to the manufacturer’s instructions. A 30 minute proteinase K (15 μl) digestion was done, and bead-beating was performed before (10 minutes) and after (5 minutes) digestion. A heating step at 90 °C for 45 min was added after proteinase K digestion and then cooled at room temperature for 10 minutes. Lysate underwent bead beating with 200 μL 0.1mm zirconia/silica beads (Biospec - #11079101z) for 10 minutes at full speed. Ethanol was added (100%, 200 μl) to the tubes followed by a short vortex mix and the whole mixture (together with beads) was loaded onto the columns in two steps. Wash and elution steps were done according to manufacturer’s instructions. Pre-heated (70°C) elution buffer (100ul) was added to the columns. Columns were incubated at 70°C for 4min before DNA was eluted. All negative controls were processed according to the exact same protocols.

#### 16S amplification and deep sequencing

Five regions of the 16S rRNA gene were amplified using 100ng DNA as an input and a set of 10 multiplexed primers (0.2 μM each primer), 0.2mM dNTPs (Larova GmbH) and 0.02U/ul of Phusion Hot Start II DNA Polymerase (Thermo Scientific #F549). Amplification was done with an initial heating step of 98 °C for 2 min, 30 cycles of 10 s at 98 °C, 15 s at 62 °C, 35 s at 72 °C followed by a final elongation step of 5 min at 72 °C. Primers used are F1-TGGCGAACGGGTGAGTAA, F2-ACTCCTACGGGAGGCAGC, F3-GTGTAGCGGTGRAATGCG,

F4-GGAGCATGTGGWTTAATTCGA, F5-GGAGGAAGGTGGGGATGAC,
R1-AGACGTGTGCTCTTCCGATCTCCGTGTCTCAGTCCCARTG,
R2- AGACGTGTGCTCTTCCGATCTGTATTACCGCGGCTGCTG,
R3- AGACGTGTGCTCTTCCGATCTCCCGTCAATTCMTTTGAGTT,
R4- AGACGTGTGCTCTTCCGATCTCGTTGCGGGACTTAACCC,
R5- AGACGTGTGCTCTTCCGATCTAAGGCCCGGGAACGTATT,

Barcodes and Illumina adaptors were then added to the amplicon with a second PCR reaction with 5 forward primers (0.2 μM each primer):

FF1-AATGATACGGCGACCACCGAGATCTACACTCTTTCCCTACACGACGCTCTTCCGATCTTGGCGAACGGGTGAGTAA,
FF2-AATGATACGGCGACCACCGAGATCTACACTCTTTCCCTACACGACGCTCTTCCGATCTACTCCTACGGGAGGCAGC,
FF3-AATGATACGGCGACCACCGAGATCTACACTCTTTCCCTACACGACGCTCTTCCGATCTGTGTAGCGGTG RAATGCG,
FF4- AATGATACGGCGACCACCGAGATCTACACTCTTTCCCTACACGACGCTCTTCCGATCTGGAGCATGTG GWTTAATTCGA,
FF5- AATGATACGGCGACCACCGAGATCTACACTCTTTCCCTACACGACGCTCTTCCGATCTGGAGGAAGGT GGGGATGAC) and one 8 nucleotide barcode-specific reverse primer (0.4 μM, RR5- CAAGCAGAAGACGGCATACGAGAT-NNNNNNNN-GTGACTGGAGTTCAGACGTGTGCTCTTCCGATCT).

The amplicon was diluted into the reaction (10-fold) and amplified with 6 cycles of 10 s at 98 °C, 15 s at 64 °C, 25 s at 72 °C’. Amplicons were then combined into sub-libraries (40-50 amplicons) and each library was purified using Qiaquick PCR purification kit (QIAGEN #28104) according to the manufacturer’s instructions. Multiple sub-libraries were then combined into the final library (100-430 amplicons) and further purified from primer dimers using Agencourt AMPure XP (Beckman Coulter #A63881) at a volume ratio of 1:0.85 (library: beads). The library (7pM) was supplemented with 15% PhiX (8pM) and sequenced on NextSeq 500 mid output (paired end 2x150) sequencers.

#### Microbiome data analysis

Sequencing data was pre-processed and mapped to the reference database as described (Cottier et al., 2018). Operational taxonomic units (OTUs) occurring only once (singletons) were removed from the entire dataset. Samples with fewer than 500 reads were removed. Alpha- (Shannon and Chao1 indices) and beta-diversity analyses (Bray-Curtis and weighted UniFrac distances) were performed in QIIME (Caporaso et al., 2010) (v1.8.0) on OTU tables that had been rarefied to 1,000 reads per sample for 10 times (Table S3). Principal Coordinate Analysis (PCoA) plots were visualized with Emperor (Vázquez-Baeza et al., 2013). The three-dimensional coordinates of the PCoA clusters from emperor results were then extracted to be plotted by python matplotlib module (v3.3.3). Only fetal and PBS derived samples were selected for the graph. Permutational Multivariate Analysis of Variance (PERMANOVA) test was done using the adonis function of vegan (v2.5.7) package in R. Table S3 contains all the samples and OTU information.

#### Data Analysis in Weizmann Institute (WIS)

Reads were demultiplexed per sample and filtered if either a) Phred score was less than 30 in more than 25% of nucleotides or b) more than three nucleotides had a Phred score of less than 10, or c) a read contained one or more ambiguous base calls. Unique reads with low counts were discarded, and reads were matched to each of the five amplified regions based on the primers’ sequences. The SMURF package was applied to combine read counts from the five regions into a coherent profiling result solving a maximum likelihood problem (Fuks et al., 2018). The Greengenes database (May 2013 version) was used as reference. To allow adding WIS results to SIgN analysis pipeline, OTU picking requiring a minimum similarity of 99% was performed over SMURF’s results. Table S4 contains all the OTU information for samples processed and sequenced at WIS.

#### Estimation of Differentially Enriched Bacterial Taxa in Gut

To explore differentially enriched bacterial taxa (OTUs at the genus level) in fetal gut as compared to PBS and Spleen or Thymus (Figures 2H and S2K), normalized read counts information of the OTUs were retrieved from Table S3. Log2 Fold Change (LOG_2_FC) of the OTUs were calculated in R (v. 4.0.3) statistical programs. The quadrant plots were made using the plot function. Each data points on the plot were sized according to the relative percentage abundance of the OTUs in Gut as compared to PBS and spleen or PBS and thymus. LOG_2_FC ≥ 2 was considered as the cut-off for OTUs which were differentially enriched in Gut whereas a Relative Percentage Abundance (RPA) of 3% is used as filtering criteria.

LOG_2_FC was calculate using the following formula.

log2FC=log2(AiBi)

Where A_i_ is the normalized read count for of the i^th^ OTU in Gut and B_i_ is the read count for the corresponding i^th^ OTU in PBS/thymus.

RPA for each OTU in gut is given by

RPAOTU=(SGut×100)(SGut+S(Spleen|Thymus)+SPBS)

$S_{Gut}$ = Counts of samples where reads exist for Gut$S_{\left(Spleen|Thymus\right)}$ = Counts of samples where reads exist for Thymus or Spleen$S_{PBS}$ = Counts of samples where reads exist for PBS

#### Bacterial Isolation and Identification

Fresh fetal tissue biopsies were taken aseptically and inoculated into pre-reduced anaerobic basal broth supplemented with 5% defibrillated horse blood, incubated anaerobically at 37°C for 48h, and then plated on anaerobic basal agar supplemented with 5% horse blood under aerobic or anaerobic conditions. Single bacterial isolates were streak-purified and then identified via standard 16S PCR and Sanger sequencing as follows. Briefly, single colonies were resuspended in 50 μL of Tris-EDTA; 1 μL of the suspension was then subjected to 1 min denaturation at 98°C, followed by 30 cycles of PCR (10 s at 98°C, 30 s at 56°C, 30 s at 72°C) and a final extension of 10 min at 72°C, using Q5® High-Fidelity DNA polymerase (NEB) and V4-V5 primers listed in Table S2. PCR products were then sequenced using V4-F as the primer, yielding sequences ≥ 330 nucleotides in length. Sequence ends were then trimmed using DNALaser Gene Seqman Pro 15 (Quality stringency: high), followed by manual inspection and further trimming to exclude regions with overlapping peaks. Chromatograms not yielding unique sequences were discarded and not processed further. Trimmed sequences were then searched against the NCBI 16S ribosomal RNA sequences (Bacteria and Archaea) database using Megablast (optimized for highly similar sequences) using default parameters. Species names of all top-scoring hits were reported (Table S5). Sequence identity was generally ≥ 97% for most of the species identified. Table S6 shows number of tissues positive for the given bacteria, and number of colonies isolated for the given bacteria, across 8 fetal donors.

#### Identification of Fetal/PBS enriched Bacterial taxa

We aligned all 1194 sampled bacterial taxa (genus level, based on the OTU information in Table S3) as identified by high-throughput 16S rRNA sequencing, according to their abundance in PBS controls. We selected top bacterial genera that were specifically present in any of the fetal tissues and top ones that were specifically enriched in PBS controls. A curated *pandas* Python library version v0.25.3 was used to extract and align the OTU reads. The bacterial taxa which showed zero signal intensity across all sample types were removed. For each sample type and bacterial identity, the mean signal intensity was calculated and plotted using the *matplotlib* (v.3.2.1) and *seaborn* (v.0.9.0) Python libraries. Bacterial genera where signal intensity in PBS was higher or equal to those in fetal samples were depicted as potential contaminants (PBS enriched taxa). Table S3 shows the OTU information for each sample. For Figure S3D, we extracted the 16S rRNA gene sequencing data for 7 fetal donors (out of 8) which were simultaneously processed for live culture-based sequencing as well. We then extracted the signal intensities for those bacterial taxa which were identified by culture-based sequencing as shown in Figure 3D. Mean signal intensities were plotted according to their abundance in fetal tissues, along with those in PBS controls (Table S3 contains the sample and OTU information for the given 7 fetal donors used in Figure S3D).

#### Scanning Electron Microscopy imaging

Fetal gut samples (14 weeks EGA) were processed for Scanning Electron Microscopy imaging under sterile conditions. Briefly, the region around mid-gut was selected for scanning and were cut open longitudinally to expose the luminal parts by using a binocular dissecting microscope and pinned down to a silicone mat in four corners. The samples (*in vitro* bacterial samples and fetal gut samples) were immediately fixed in 2.5% glutaraldehyde overnight at 4°C. Phosphate buffer solution (1X PBS) was used to wash the overnight fixed samples (2 changes, 20 minutes each) and the samples were kept in PBS. For bacterial cultures, bacteria isolated from 18 weeks EGA fetal lungs and gut (OD_600_ = 0.1) were co-incubated with nitrocellulose membrane in Luria-Bertani broth overnight at 37°C to allow bacteria to attach the membranes, prior to fixation. The samples were post-fixed in 1% osmium tetroxide and dehydrated by using ascending concentrations of ethanol. After which, the samples were critical-point-dried, stuck on carbon stubs and coated with 25 nm of gold. The samples were imaged on a Field Emission Electron Microscope (EM) at a voltage of 20 kV at Electron Microscopy Unit at the National University of Singapore.

#### RNA *in situ* hybridization

Formalin-fixed paraffin-embedded (FFPE) slides were prepared from FFPE fetal tissue blocks. The tissue samples with a thickness of 2 microns were mounted onto glass slides and stored at room temperature. Slides were stained using the RNAscope® Multiplex Fluorescent Reagent Kit v2 Assay Kit (Advanced Cell Diagnostics) following manufacturer’s protocol. The slides were baked in a dry oven for 1 hour at 60°C. Following steps included deparaffinizing the slides using fresh xylene (2 washes for 5 minutes each) and 100% ethanol (2 washes for 2 minutes each). The slides were then treated with RNAscope® Hydrogen Peroxide for 10 minutes at room temperature. Meanwhile, 200 mL of RNAscope® 1X Target Retrieval Reagent was poured in a slide holder and kept in a beaker filled with water on a hot plate to boil. The slides were washed and put in the slide holder for 15 minutes at 99°C for target retrieval. A hydrophobic barrier was drawn around the tissue using ImmEdge hydrophobic barrier pen. Few drops of RNAscope® Protease Plus was added onto the tissues and incubated for 30 minutes at 40°C. RNAscope probes from ACDbio was used in this experiment; EB-16 s-rRNA (Cat# 464461) in C1 channel and EPCAM (Cat# 310281) in C3 channel. The probe mix was added onto the slide and probe hybridization was performed for 2 hours at 40°C. The slides were then stored in 5X SSC overnight and amplification steps were performed the following day.

Opal 520 and Opal 690 dyes (Perkin Elmer, Cat# NEL821001KT) were selected for EB-16 s-rRNA and EPCAM probes respectively. The Opal dye dilution optimized was 1:500 for all samples except 1:250 dilution for liver samples (EPCAM probe only). The slides were mounted using a drop of ProLong Diamond Antifade Mountant (Thermofisher, Cat#: P36970). The slides were imaged by Vectra® Polaris Automated Quantitative Pathology Imaging System. The images were edited using inForm® image analysis software and color intensity for EB-16 s-rRNA was set to 150% (green) while EPCAM was set to 120% (white). The whole slide scans of Hematoxylin and eosin stained slides were imaged using Phenochart image analysis software.

For image quantification, Imagej and Cell Profiler softwares were used. Using Imagej software, the images were split into single channel greyscale images. These greyscale images were added into Cell Profiler software and analyzed using a published pipeline (Erben et al., 2018). The threshold correction factor in the pipeline was in the range of 1-4 for EB-16 s-rRNA and 1.7-2.5 for EPCAM.

#### *In vitro* syngeneic T cell expansion assay

DC and T cells from fetal Mesenteric Lymph Node (mLN) were isolated by fluorescence activated cell sorting (FACS) using BD FACSAria IV (BD Biosciences). T cells were isolated by negative selection, gating for lineage negative (lineage- CD19/20/56) and HLA-DR negative cells, with upto ∼80 percent purity. HLA-DR^+^ and CD14^-^ cells were selected for DC isolation. Fetal bacteria (Lactobacilli and Salmonella) were isolated and cultured as indicated above, in 5% defibrillated horse blood at 37°C for 48h. Culture stocks were made and snap frozen at −80°C. At the day of experiment, frozen vials were thawed and bacteria were washed with sterile PBS followed by heat inactivation at 65°C for 20 minutes, with intermittent vortex mixing. Flow sorted DC were plated in a 96 well plate at the density of 1000 cells per well in the DC specific media: IMDM, 10% human serum AB, 1% v/v sodium pyruvate, 2mM glutamine, and 200ng/ml human FLT3 ligand (cat no. 130-096-479, Milteny Biotec). Fetal bacteria (heat-killed) were added to DC with an MOI of 1:10 (10,000 bacteria per 1000 DC) and incubated for 8 hours at 37°C. T cells were labeled with CellTrace Violet (CTV) dye and a small portion was analyzed by flow cytometry for dye incorporation and cell viability. DC were gently washed with sterile PBS and CTV labeled T cells were added to each well in 1:10 ratio of DC:T cells (10,000 T cells per 1000 DC), and incubated for 6 days in T cell media: IMDM, 10% human serum AB, 1% v/v sodium pyruvate, 2mM glutamine, 1% Pen-Step solution (antibiotic) (Hegazy et al., 2017). Post 6 days culture, T cells were harvested and stained with fluorescent antibodies followed by flow-cytometry analysis using BD-LSR-V (BD biosciences) and data were analyzed using FlowJo software.

For PFA induced DC fixation: DC were first infected with fetal bacteria for 8 hours, followed by fixation using 1% PFA for 10 minutes. The PFA was then removed after 10 mins and the reaction was quenched with 200mM glycine. Cells were washed twice with sterile PBS and RPMI respectively. Rest of the process of T cell addition and incubation proceeds as explained earlier.

For PTI induced blocking of cytokine release: Post bacterial priming of DC for 8 hours, cells were incubated with a combination of Brefeldin A and Monensin (Protein Transport Inhibitors) for 2 hours. The cells were then washed twice with sterile PBS and RPMI respectively. Rest of the process of T cell addition and incubation proceeds as explained earlier.

#### Statistical analysis

Statistical analysis was performed in GraphPad Prism 7 using the specific tests indicated in the respective figure legends. The number of samples used for each analysis (n) is indicated in the respective figure or legend.

## Supplemental information

Table S1. Human samples information, related to Figures 1, 2, 3, S1, S2, and S3

Table S2. Sequencing primers, related to Figures 2, 3, S2, and S3

Table S3. 16S rRNA sequencing reads and extended OTU information (metadata), related to Figures 2, 3, S2, and S3

Table S4. Sequencing reads for samples at WIS, related to Figures 2 and S2

Table S5. Culture sequenced bacteria information, related to Figures 3 and S3

Table S6. Bacterial colony isolation from fetal tissue cultures, related to Figures 3 and S3
